# Interpretable nonconvex submodule clustering algorithm using *ℓ_r_*-induced tensor nuclear norm and *ℓ_2,p_* column sparse norm with global convergence guarantees

**DOI:** 10.1371/journal.pone.0339534

**Published:** 2026-01-02

**Authors:** Ming Yang, Shumao Han, Linglong Chen, Jiayi Wang

**Affiliations:** School of Mathematical Sciences, Harbin Engineering University, Harbin, China; Michigan State University, UNITED STATES OF AMERICA

## Abstract

Tensor-based subspace clustering algorithms have garnered significant attention for their high efficiency in clustering high-dimensional data. However, when dealing with 2D image data, traditional vectorization operations in most algorithms tend to undermine the correlations of higher-order tensor terms. To tackle this limitation, this paper proposes a non-convex submodule clustering approach (2D-NLRSC) that leverages sparse and low-rank representations for 2D image data. An $\ell_{r}$-induced tensor nuclear norm is introduced to approximate the tensor rank precisely. Instead of vectorizing each 2D image, the framework arranges samples as lateral slices of a third-order tensor. It employs the *t*-product operation to generate an optimal representation tensor with low-rank constraint. The proposed method combines $\ell_{q}$-norm induced clustering awareness with laplacian regularization to obtain a representation tensor with a diagonal structure. Additionally, 2D-NLRSC incorporates the $\ell_{2,p}$-norm as a regularization term, taking advantage of its excellent invariance, continuity, and differentiability. Experimental results on real image datasets validate the superior performance of the 2D-NLRSC model.

## 1 Introduction

High-dimensional data often resides within lower-dimensional subspaces, revealing underlying subspace structures [1]. This insight underpins subspace clustering, which assumes that data points are sampled from multiple subspaces, with each point being linearly representable by a subset of points within the same subspace. The objective of subspace clustering is to divide data into clusters, where the points within each cluster lie in the same subspace. To address these challenges, a range of subspace clustering techniques have been proposed. Traditional subspace clustering methods, for example, Sparse Subspace Clustering (SSC) [2] and Low-Rank Representation (LRR) [3], have been extensively employed to partition data into multiple, potentially overlapping linear subspaces, thereby minimizing redundancy. These techniques have proven highly effective across various applications involving high-dimensional data, such as image clustering and temporal video segmentation [3]. Among the most prominent techniques, spectral clustering-based approaches have gained substantial traction over the past decade. These approaches involve constructing an affinity matrix and applying algorithms such as K-means or Normalized Cuts (Ncut). The versatility of these algorithms in diverse clustering tasks has been a key factor in their growing popularity. Recently, several works have focused on addressing performance degradation caused by noise in multi-view clustering. For example, Zhou et al. [4] proposed the RTOSNMF method, which tackles the issues of noise interference and insufficient utilization of inter-layer relationships in multi-layer network community detection by performing denoising via linear separation and the $\ell_{2,1}$-norm, as well as exploring inter-layer relationships through nuclear norm-constrained low-rank property. Che et al. [5] put forward a robust multi-view clustering method based on weighted low-rank tensor approximation and noise separation: on one hand, it designs differentiated constraints for different types of noise to achieve fine grained noise elimination; on the other hand, it efficiently explores high-order correlations among multiple views through weighted low-rank tensor modeling. Xie et al. [6] reformulated the min-cut problem as a bi-bounded constraint problem and developed an algorithm suitable for size-constrained min-cut scenarios and generalizable to broader bi-bounded nonlinear optimal transport problems. Shi et al. [7] proposed an optimal transport framework with both upper and lower bound constraints to enhance clustering and classification by better capturing structural relationships in data.

However, conventional subspace clustering methods encounter challenges when dealing with high-dimensional tensor data [2,3]. This challenge lies at the core of the current research. These techniques often lack robust theoretical guarantees for clustering accuracy, particularly due to the high dimensionality of the data, which requires substantial time and memory resources. Our framework addresses this gap by providing a robust, tailored solution for image data clustering.

When handling imaging data, traditional subspace clustering methods often vectorize data samples, enabling the application of algorithms designed for vectorial data. While effective in some cases, this vectorization process disrupts the intrinsic multidimensional structure of images, thereby reducing the reliability of subsequent analysis. It increases the dimensionality of the data, exacerbating the curse of dimensionality, and it also destroys the inherent high-dimensional structure of the data. To address this limitation, various works have leveraged multilinear algebra tools to preserve and exploit the spatial characteristics of imaging data [8–10]. One significant innovation in this domain is the *t*-product, a matrix-analogous multiplication operation for third-order tensors that improves the exploitation of their internal structure. In this framework, the representation matrices from various views are arranged into slices of a three-dimensional tensor [11–13]. Utilizing operations from multilinear and abstract algebra enables the more effective exploration of third-order tensor properties. The introduction of the *t*-product simplifies tensor-tensor multiplication, enabling more efficient and accurate modeling of image data [14,15].

For instance, Wu [16] proposed a tensor-based submodule clustering method for 2D imaging data, which organized samples as lateral slices of third-order tensors via *t*-product, integrated low-rank constraints, manifold regularization, and a unified ADMM-spectral clustering framework with nonlinear extensions. Francis et al. [17] developed a robust unsupervised tensor subspace clustering method using $\ell_{1/2}$-regularized tensor nuclear norm (TNN) to preserve geometric structures, enable slice-wise sparse-low-rank decomposition for noise removal. Madathil et al. [18] introduced a noise-robust tensor clustering approach via reweighted nuclear norms for enhanced low-rank representation, $\ell_{2,1}$-norm structured sparsity, and explicit noise separation, achieving high accuracy under severe corruption. Francis et al. [19] proposed a single-stage framework for incomplete imaging data, unifying tensor clustering (via sparse *t*-linear combinations with mode-3 low-multirank constraints) and missing-data reconstruction (through low-rank lateral slice approximations), thus preserving spatial structure.

Inspired by the above, we adopt the *t*-product, based on circular convolution, to model the dynamic characteristics of consecutive image sequences. Using the *t*-product, image data samples are grouped into a third-order tensor and represented by our proposed tensor low-rank model, built around the union of free submodules. The resulting affinity information is then utilized for final clustering.

By combining *t*-product operations and tensor factorization, we extend the traditional LRR clustering method to accommodate multi-view data. This paper employs sparse and low-rank representations, similar to those mentioned above. However, it incorporates the latest developments in sparse coding to propose a highly interpretable $\ell_{r}$-induced tensor nuclear norm. The optimization of this norm is based on the studies of non-convex $\ell_{r}$-norm by Candes et al. [20], Zuo et al. [21], and Zha et al. [22], combining sparsity and low-rank properties.

Peng et al. [23] proposed a novel NMF algorithm with an $\ell_{2,\log}$-(pseudo) norm applied to the factor matrix to enforce sparse properties. Inspired by this novel column-sparse norm and influenced by [22], we naturally generalize the above definition along the frontal slice direction to the tensor-based $\ell_{p}$-induced sparse approximation, thereby proposing an $\ell_{2,p}$-(pseudo) norm.

A dissimilarity matrix *M* is constructed to impose constraints on the frontal slices of the representation tensor. Specifically, elements with smaller values (indicating higher similarity) in the dissimilarity matrix are used to inversely constrain the corresponding positions in the slices of the representation tensor to take on larger values. This approach enhances the representation tensor, yielding a more distinct cluster structure. We show that the nonconvex $\ell_{q}$-norm, combined with our cluster-aware construction, effectively captures the block structure of the self-representation tensor 𝒵. With an appropriate value of *q*, the $\ell_{q}$-norm offers unique advantages in sparsity representation. In certain scenarios, selecting an appropriate value of *q* enables the $\ell_{q}$-norm to achieve unique advantages in sparsity representation.

The key contributions of our paper are as follows.

- The proposed 2D-NLRSC algorithm leverages the submodular self-expression property to address the issue in traditional tensor subspace clustering where vectorization of 2D images destroys higher-order tensor correlations.It directly constructs image samples as lateral slices of a third-order tensor, effectively avoiding structural information loss caused by vectorization.
- The $\ell_{r}$-induced tensor nuclear norm is employed to accurately approximate the tensor rank, and combined with *t*-product operations to construct an optimal representation tensor under low-rank constraints, resolving the problem that traditional convex tensor nuclear norms fail to precisely characterize tensor low-rank structures.
- The $\ell_{2,p}$-norm is incorporated into 2D-NLRSC as a regularization term, utilizing its excellent invariance, continuity, and differentiability to enhance the model’s resilience to outliers while optimizing the selection of representative data points to reduce redundancy. Additionally, the clustering-aware property of the $\ell_{q}$-norm is combined with Laplacian regularization to guide the representation tensor in forming block-diagonal structures consistent with clustering objectives.
- An efficient alternation direction method of multipliers (ADMM) algorithm is proposed, with its convergence rigorously proven based on the Karush-Kuhn-Tucker (KKT) conditions. Experiments conducted on real-world datasets validate the effectiveness of the proposed method.

The remainder of this paper is organized as follows. Sect 2 introduces the preliminaries. Sect 3 reviews the related work. Sect 4 presents the proposed 2D-NLRSC method and its optimization procedure. Experimental results are reported in Section 5, while Sect 6 provides the convergence analysis. Finally, Sect 7 concludes the paper.

## 2 Notations and preliminary considerations

Here, the relevant definitions and preliminary notations for the variables are first presented. For the sake of conciseness, the related notations are summarized in Table 1.

**Table 1:** Notations.

Notation	Definition
x	Vector **x**
*X*	Matrix *X*
𝒳	Tensor 𝒳
*N*	Number of samples
tr(X)	Trace of *X*
${𝑋}_{i,j}$	The (*i*,*j*)-th element of *X*
${𝒳}_{i,j,k}$	The (*i*,*j*,*k*)-th element of 𝒳
${𝒳}^{\left(v\right)}$	The *v*-th frontal slice of 𝒳
${𝐵}^{\left(v\right)}$	Adjacency matrix
${𝐷}^{\left(v\right)}$	Degree matrix
*L*	Laplacian matrix
𝒞,𝒬,𝒯	Auxiliary variables
$S\in {\mathbb{R}}^{N\times N}$	Affinity matrix
$M\in {\mathbb{R}}^{N\times N}$	Dissimilarity matrix
$\|𝒳{\|}_{F}=\sqrt{\sum_{i,j,k}|{𝒳}_{i,j,k}{|}^{2}}$	Frobenius norm of 𝒳
$\|𝒳{\|}_{2,1}=\sum_{i,j}\|𝒳(i,j,:){\|}_{2}$	$\ell_{2,1}$-norm of 𝒳
$\|𝒳{\|}_{2,p}=\|\text{unfold}(𝒳){\|}_{\ell_{2,p}}$	$\ell_{2,p}$-norm of 𝒳
$\|𝒳{\|}_{⊛r}=\sum_{k=1}^{n_{3}}\sum_{i=1}^{\min(n_{1},n_{2})}|\hat{\Sigma }(i,i,k){|}^{r}$	$\ell_{r}$-induced TNN of 𝒳

**Definition 1.** (*t*-product [10]): Let $𝒜\in {\mathbb{R}}^{n_{1}\times n_{2}\times n_{3}}$ and $ℬ\in {\mathbb{R}}^{n_{2}\times n_{4}\times n_{3}}$. Then the *t*-product 𝒞=𝒜*ℬ is a tensor of size $n_{1}\times n_{4}\times n_{3}$, i.e.,

𝒞=𝒜*ℬ=fold(bcirc(𝒜)·unfold(ℬ)). (1)

where bcirc(𝒜) is defined as

$$\text{bcirc}(𝒜)=[\begin{array}{cccc} A^{\left(1\right)} & A^{\left(n_{3}\right)} & \ldots & A^{\left(2\right)}\\ A^{\left(2\right)} & A^{\left(1\right)} & \ldots & A^{\left(3\right)}\\ \vdots & \vdots & \ddots & \vdots \\ A^{\left(n_{3}\right)} & A^{\left(n_{3}-1\right)} & \ldots & A^{\left(1\right)} \end{array}].$$ (2)

The operator unfold(ℬ) and its corresponding inverse operator fold(ℬ) are defined as

$$\text{unfold}(ℬ)=[\begin{array}{c} {ℬ}^{\left(1\right)}\\ {ℬ}^{\left(2\right)}\\ \vdots \\ {ℬ}^{\left(n_{3}\right)} \end{array}],\;\text{fold}(\text{unfold}(ℬ))=ℬ.$$ (3)

**Theorem 1.** (*t*-SVD [24]): For $𝒜\in {\mathbb{R}}^{n_{1}\;\times \;n_{2}\;\times \;n_{3}}$, the *t*-SVD of 𝒳 is given by $𝒳=𝒰\;*\;\Sigma \;*\;{𝒱}^{⊤}$ where $𝒰\in {\mathbb{R}}^{n_{1}\;\times \;n_{1}\times n_{3}}$ and $𝒱\in {\mathbb{R}}^{n_{2}\;\times \;n_{2}\;\times \;n_{3}}$ are orthogonal tensors. Σ is an *f*-diagonal tensor, and * denotes the *t*-product.

**Definition 2.** (tensor $\ell_{2,p}$-norm [25]) For a matrix $𝐻\in {\mathbb{R}}^{n_{1}\times n_{2}}$, the $\ell_{2,p}$-norm is defined as

$$\|𝐻{\|}_{2,p}=\sum_{k=1}^{n_{2}}{\left(\sum_{l=1}^{n_{1}}{𝐻}_{lk}^{2}\right)}^{\frac{p}{2}}.$$ (4)

where p∈(0,1]. Particularly, considering *p* = 1, the $\ell_{2,p}$-norm reduces to the $\ell_{2,1}$-norm:

$$\|𝐻{\|}_{2,1}=\sum_{k=1}^{n_{2}}\sqrt{\sum_{l=1}^{n_{1}}{𝐻}_{lk}^{2}}.$$ (5)

We then extend matrix $\ell_{2,p}$-norm to tensor $\ell_{2,p}$-norm as follows

$$\|𝒜{\|}_{2,p}=\|\text{unfold}(𝒜){\|}_{2,p}.$$ (6)

## 3 Related work

Consider a data set $𝕏=\{{𝑋}_{k}\in {\mathbb{R}}^{n_{1}\times n_{3}}|k=1,\ldots ,N\}$ that contains *N* images partitioned into *m* categories. Conventional subspace clustering pipelines vectorize each image ${𝑋}_{k}$ into a flattened vector ${𝐱}_{k}\in {\mathbb{R}}^{n_{1}n_{3}}$, constructing a data matrix $𝑋=[{𝐱}_{1},{𝐱}_{2},\ldots ,{𝐱}_{N}]$. These vectors are hypothesized to reside near a union of *m* low-dimensional subspaces embedded in ${\mathbb{R}}^{n_{1}n_{3}}$. The clustering objective is to group data points based on their intrinsic subspace affiliations.

Although the above vectorization approach has demonstrated excellent performance in numerous applications, it fails to account for the spatial structure of images. Additionally, these methods, which are typically used to represent the linear combinations of samples in subspaces, are unable to capture shifted copies within submodules. In contrast, the *t*-product [10] offers a novel algebraic approach that generalizes matrix multiplication to third-order tensors.

### 3.1 Linear algebra with the *t*-product

To preserve the spatial structure of data, *N* oriented matrices of size $n_{1}\;\times \;1\;\times \;n_{3}$ are stacked into a third-order tensor $𝒴\in {\mathbb{R}}^{n_{1}\;\times \;N\;\times \;n_{3}}$. Here, ${𝕂}_{n_{3}}$ denotes the set of tube fibers of dimension $1\;\times \;1\;\times \;n_{3}$, and ${𝕂}_{n_{3}}^{n_{1}}$ denotes the set of oriented matrices of size $n_{1}\;\times \;1\;\times \;n_{3}$. The goal is to define a multiplication operation between tube fibers, enabling “linear” combinations of oriented matrices in which the coefficients are themselves tube fibers rather than scalar values.

Following [26], the set ${𝕂}_{n_{3}}^{n_{1}}$ can be regarded as a *module* over the ring ${𝕂}_{n_{3}}$. From this viewpoint, the *t*-product provides a natural generalization of matrix multiplication to third-order tensors, where the multiplication between elements is replaced by tube fiber multiplication, and the addition remains elementwise.

### 3.2 Representation of high-dimensional image data

Given an image matrix $X\in {\mathbb{R}}^{n_{1}\times n_{3}}$, we embed it into a third-order tensor by orienting it along the third mode

$$\vec{𝒳}=reshape(X,\;n_{1}\times 1\times n_{3})\in {𝕂}_{n_{3}}^{n_{1}},$$ (7)

where each lateral slice $\vec{𝒳}(i,1,:)$ is a *tube fiber* of length *D*. Let $𝒳=[\;{\vec{𝒳}}_{1},{\vec{𝒳}}_{2},\ldots ,{\vec{𝒳}}_{N}\;]\in {\mathbb{R}}^{n_{1}\times N\times n_{3}}$ denote the collection of *N* such oriented tensors.

In the *t*-product framework, ${𝕂}_{n_{3}}^{n_{1}}$ is viewed as a free module of rank *n*_1_ over the ring ${𝕂}_{n_{3}}$. Given a generating set (dictionary) $\{{\vec{𝒟}}_{i}{\}}_{i=1}^{s}\subset {𝕂}_{n_{3}}^{n_{1}}$, any $\vec{𝒳}\in {𝕂}_{n_{3}}^{n_{1}}$ admits a *t*-linear representation

$$\vec{𝒳}=\sum_{i=1}^{s}{\vec{𝒟}}_{i}*{\vec{𝑡}}_{i},\;{\vec{𝑡}}_{i}\in {𝕂}_{n_{3}}.$$ (8)

For computational purposes, let $𝒟=[\;{\vec{𝒟}}_{1},\ldots ,{\vec{𝒟}}_{s}\;]\in {\mathbb{R}}^{n_{1}\times s\times n_{3}}$ and $\vec{𝑡}=[\;{\vec{𝑡}}_{1},\ldots ,{\vec{𝑡}}_{s}\;{]}^{⊤}\in {\mathbb{R}}^{s\times 1\times n_{3}}$. Then the above expression can be compactly written as

$$\vec{𝒳}=𝒟*\vec{𝑡}.$$ (9)

Applying the discrete Fourier transform (DFT) along the third mode yields

$${\hat{𝐗}}^{\left(k\right)}={\hat{𝐃}}^{\left(k\right)}\;{\hat{𝐭}}^{\left(k\right)},\;k=1,\ldots ,n_{3},$$ (10)

where ${\hat{𝐗}}^{\left(k\right)}\in {\mathbb{C}}^{n_{1}}$, ${\hat{𝐃}}^{\left(k\right)}\in {\mathbb{C}}^{n_{1}\times s}$, and ${\hat{𝐭}}^{\left(k\right)}\in {\mathbb{C}}^{s}$ denote the *k*-th frontal slices in the Fourier domain. This block-diagonal structure allows independent processing of each frequency slice, retaining spatial structure while enabling efficient computation.

By preserving the tensor structure of the original images, this representation mitigates the loss of spatial correlations caused by vectorization, and naturally supports linear modeling in the tensor algebra framework.

### 3.3 Submodule clustering by sparse and low-rank representation

Based on this theoretical framework, the sparse submodule clustering (SSmC) algorithm, as introduced in [26], can be expressed as the subsequent optimization problem

$$\min_{𝒵}\|𝒵{\|}_{F1}+\lambda_{1}\|𝒵{\|}_{FF1}+\lambda_{2}\|𝒴-𝒴*𝒵{\|}_{F}^{2}\;\text{s.t. }z_{iik}=0,\forall i=1,2,\ldots ,N,\forall k=1,2,\ldots ,n_{3},$$ (11)

where $𝒵\in {\mathbb{R}}^{N\times N\times n_{3}}$ denotes the representation tensor.

Inspired by the concept that images from different free submodules [27] should exhibit low correlation, Wu et al. proposed the structurally constrained low-rank submodular clustering (SCLRSmC) in [14]. The learning process for this method can be expressed as

$$\min_{𝒵}\|𝒵{\|}_{TNN}+\lambda_{1}\sum_{k=1}^{n_{3}}{\left\|𝐵⊙{𝒵}^{\left(k\right)}\right\|}_{\ell_{1}}+\lambda_{2}{\left\|𝒴-𝒴*𝒵\right\|}_{F}^{2},$$ (12)

where $𝐵\in {\mathbb{R}}^{N\times N}$ is a predefined weight matrix related to the data, and ⊙ represents the Hadamard product.

Additionally, Wu [28] proposed an online low-rank tensor subspace clustering (OLRTSC) algorithm based on the nonconvex reformulation of tensor low-rank representation (TLRR) and the *t*-SVD framework, aiming to recover efficiently and cluster tensor data. This approach significantly reduces computational complexity and storage costs, handles dynamic data, and extends to scenarios with missing data. He also introduced an outlier-robust tensor low-rank representation (OR-TLRR) method, which simultaneously performs outlier detection and tensor data clustering within the *t*-SVD framework [29]. Research shows that *t*-SVD effectively reduces data dimensionality, extracts key features, and enhances data processing efficiency.

Although the above algorithms have shown promising results on real datasets, approximating tensor rank with the nuclear norm remains an imprecise problem. Since the nuclear norm treats every eigenvalue equally and penalizes noise components, it can lead to a suboptimal representation tensor. Therefore, this approach still requires improvement.

Recently, Jobin et al. [17,30] proposed a multi-view data clustering framework based on nonconvex low-rank tensor approximation

$$\min_{Z}{\left\|𝒵\right\|}_{⊛\frac{1}{2}}+\lambda_{1}\sum_{k=1}^{V}{\left\|𝒫\circ {𝒵}^{\left(k\right)}\right\|}_{\frac{1}{2}}^{\frac{1}{2}}+\lambda_{2}{\left\|𝒳-𝒳*𝒵\right\|}_{F}^{2}.$$ (13)

**Definition 3.** ($\ell_{\frac{1}{2}}$-induced tensor nuclear norm): Consider a tensor, $𝒳\in {\mathbb{R}}^{n_{1}\times n_{2}\times n_{3}}$ with *t*-SVD $𝒳=𝒰*\Sigma *{𝒱}^{⊤}$, then its $\ell_{\frac{1}{2}}$-induced tensor nuclear norm can be expressed as

$${\left\|𝒳\right\|}_{⊛\frac{1}{2}}=\sum_{k=1}^{n_{3}}\sum_{i=1}^{\min(n_{1},n_{2})}\sqrt{\left|\hat{\Sigma }(i,i,k)\right|}.$$ (14)

Notably, this framework employs the $\ell_{\frac{1}{2}}$-induced tensor nuclear norm (TNN) as a low tensor rank constraint, which enhances the low-rank property of the representation tensor. However, Zuo et al. [21] demonstrated in the sparse coding context that $\frac{1}{2}$ is not necessarily optimal, and a general r∈(0,1] should be considered to balance rank sparsity and numerical stability. The generic $\ell_{r}$-norm minimization problem takes the form

$$\hat{𝐱}=\arg\min_{𝐱}\frac{1}{2}\|𝐱-𝐲{\|}_{2}^{2}+\lambda \|𝐱{\|}_{\ell_{r}}^{r},$$ (15)

where **y** is the input vector (e.g., a singular value vector), and $\|𝐱{\|}_{\ell_{r}}^{r}=\sum_{i}|x_{i}{|}^{r}$ with 0<r≤1.

Problem (15) can be efficiently solved by the *generalized soft-thresholding* (GST) algorithm [21], which alternates between a gradient descent step and a generalized shrinkage step. Specifically, given **y**, the GST update is

$${𝐱}^{\left(k+1\right)}={𝕋}_{r}^{GST}\left(𝐲;\lambda \right),$$ (16)

where ${𝕋}_{r}^{GST}$ is applied element-wise:

$${𝕋}_{r}^{GST}(y;\lambda )=\left\{ \begin{array}{cc} 0, & |y|\leq \tau_{r}(\lambda ),\\ sign(y)\;z^{*}, & |y|>\tau_{r}(\lambda ), \end{array}\right.$$ (17)

with threshold $\tau_{r}(\lambda )=\left(2\lambda (1-r\right){)}^{\frac{1}{2-r}}+\lambda r\left(2\lambda (1-r\right){)}^{\frac{r-1}{2-r}}$ and z^*^ being the positive root of $z-y+\lambda rz^{\;r-1}=0$.

As pointed out by Zha et al. [22], $\ell_{r}$ minimization with *r*<1 can more aggressively promote sparsity (and thus low rank) compared to the convex *r* = 1 case, often leading to better empirical performance in subspace clustering tasks.

Inspired by the above, we introduce the $\ell_{r}$-induced tensor nuclear norm to enforce low-rank structure in the tensor. Unlike [30], our method allows adjusting the value of *r* between 0 and 1, enabling a more flexible and effective low-rank representation of the tensor.

**Definition 4.** ($\ell_{r}$-induced tensor nuclear norm): Consider a tensor $𝒳\in {\mathbb{R}}^{n_{1}\times n_{2}\times n_{3}}$ with *t*-SVD $𝒳=𝒰*\Sigma *{𝒱}^{⊤}$. It’s $\ell_{r}$-induced TNN can be defined as

$$\|𝒳{\|}_{⊛r}=\sum_{k=1}^{n_{3}}\sum_{i=1}^{\min(n_{1},n_{2})}|\hat{\Sigma }(i,i,k){|}^{r}.$$ (18)

where r∈(0,1], $𝒰\in {\mathbb{R}}^{n_{1}\times n_{1}\times n_{3}}$ and $𝒱\in {\mathbb{R}}^{n_{2}\times n_{2}\times n_{3}}$ are the orthogonal tensors, and Σ is an *f*-diagonal tensor whose frontal slices contain diagonal matrices, and $\hat{\Sigma }$ denotes its Fourier transform.

Xie et al. proposed a nonconvex tensor multi-view clustering framework [25], which introduces a novel column-sparse norm instead of the squared Frobenius norm for 𝒳−𝒳*𝒵: the $\ell_{2,p}$- norm with p∈(0,1]. This norm exhibits properties such as invariance, continuity, and differentiability. Inspired by [25], this paper applies the $\ell_{2,p}$- norm to 2D images.

## 4 Proposed method

### 4.1 Problem formulation and objective function

Recognizing that most conventional clustering methods rely on vectorized data representations and often overlook the intrinsic structure of image data, we focus on leveraging tensor representations, particularly for 2D images. Consequently, we propose a novel image submodule clustering method that preserves and leverages the inherent structure of image data to achieve more robust and reliable clustering outcomes.

For the 2D image samples *X* mentioned above, rather than vectorizing each image as in traditional subspace clustering methods, the images are arranged side by side to form a tensor $𝒳\in {\mathbb{R}}^{n_{1}\times n_{3}\times N}$. This tensor is then rotated to a dimension of $n_{1}\times N\times n_{3}$, where $n_{1}\times n_{3}$ denotes the size of the images and *N* indicates the number of images. Each image sample is represented using *t*-linear combinations. To obtain the optimal low-rank representation tensor $𝒵\in {\mathbb{R}}^{N\times N\times n_{3}}$, we apply an $\ell_{r}$-induced tensor nuclear norm on 𝒵. We add a sparse norm, the $\ell_{2,p}$-norm, where p∈(0,1], to strengthen the robustness of our approach. A slice-wise Laplacian regularization term centered around each image is also introduced to retain local details and expose nonlinear structures in high-dimensional space. This regularization guarantees consistent representations of data points within each image in the global space, enhancing overall efficiency. Therefore, the combination of sliced Laplacian, $\ell_{2,p}$ regularization, and tensor $\ell_{r}$-induced TNN can be represented as follows

$$\min_{𝒵,ℰ}\|𝒵{\|}_{⊛r}+\lambda_{1}\sum_{v=1}^{n_{3}}tr({𝒵}^{\left(v\right)}{𝐿}^{\left(v\right)}({𝒵}^{\left(v\right)}{)}^{⊤})+\lambda_{2}\|ℰ{\|}_{2,p}\;\text{s.t.}\;𝒳=𝒳*𝒵+ℰ,$$ (19)

where $𝒳\in {\mathbb{R}}^{n_{1}\times N\times n_{3}}$, $𝒵\in {\mathbb{R}}^{N\times N\times n_{3}}$, $ℰ\in {\mathbb{R}}^{n_{1}\times N\times n_{3}}$, and ${𝐿}^{\left(v\right)}={𝐷}^{\left(v\right)}-{𝐵}^{\left(v\right)}$ is the Laplacian matrix of ${𝐵}^{\left(v\right)}$, the adjacency matrix of a *k*-nearest neighbor (KNN) graph. It can be constructed by

$$B_{i,j}^{\left(v\right)}=\left\{ \begin{array}{cc} \exp\left(-\frac{\|\overset{―}{𝒳}(:,:,i)-\overset{―}{𝒳}(:,:,j){\|}_{F}^{2}}{2\sigma^{2}}\right), & 𝒳(:,j,:)\in {𝒩}_{\delta }(𝒳(:,i,:))\text{ or }\\ & 𝒳(:,i,:)\in {𝒩}_{\delta }(𝒳(:,j,:))\\ 0, & \text{otherwise}. \end{array}\right.$$ (20)

Here, ${𝒩}_{\delta }(𝒳(:,i,:))$ denotes the δ-neighborhood of 𝒳(:,i,:) under a given distance metric ξ. Specifically, we define $\xi \left(𝒳(:,i,:),𝒳(:,j,:)\right)={\left\|\overset{―}{𝒳}(:,i,:)-\overset{―}{𝒳}(:,j,:)\right\|}_{F}$, where $\overset{―}{𝒳}$ is derived by normalizing each lateral slice of 𝒳 such that ${\left\|\overset{―}{𝒳}(:,i,:)\right\|}_{F}=1$ for all i=1,2,…,N.

The degree matrix ${𝐷}^{\left(v\right)}$ is a diagonal matrix where the *i*-th diagonal element is computed as ${𝐷}_{i,i}^{\left(v\right)}=\sum_{j=1}^{N}{𝐵}_{i,j}^{\left(v\right)}$. Relevant details can be found in [25]. Furthermore, the suitable block diagonal structure representing tensors is beneficial for clustering multi-view data, enhancing the algorithm’s performance. In multi-view data, objects that belong to the same submodule exhibit a strong correlation, while objects from different submodules show relatively lower correlation [14]. To enforce a diagonal structure on 𝒵, we prioritize images that exhibit a lower correlation between those belonging to different submodules. This correlation between different data points can be captured by the inconsistency weighted matrix $𝑀\in [0,1{]}^{N\times N}$. The elements of *M* are defined as

$$M_{ij}=1-\exp\left(-\frac{1-|\langle \overset{―}{𝒳}(:,i,:),\overset{―}{𝒳}(:,j,:)\rangle |}{\sigma }\right).$$ (21)

where $\overset{―}{𝒳}$ represents the tensor that is acquired by normalizing every lateral slice of 𝒳 in a way that $\|\overset{―}{𝒳}(:,i,:){\|}_{F}=1$ for i=1,2,…,N, and σ is typically taken as the empirical average of all $1-|\langle \overset{―}{𝒳}(:,i,:),\overset{―}{𝒳}(:,j,:)\rangle |$.

**Fig 1:**
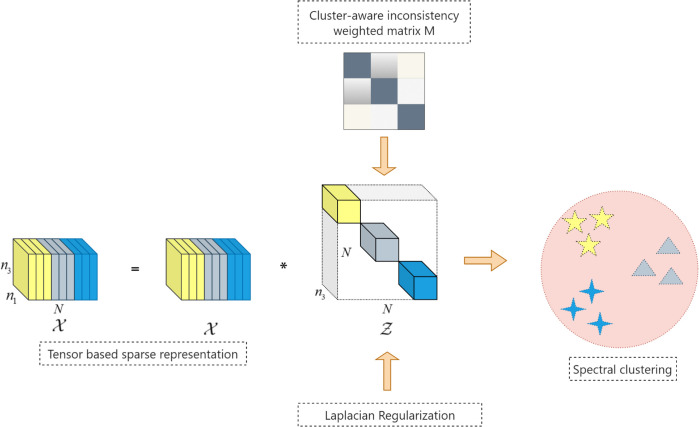
The framework of proposed 2D-NLRSC.

Unlike (12), we obtain a sparser solution by substituting the $\ell_{1}$-norm with the$\ell_{q}$-norm. By integrating all the above, our algorithm can be summarized as follows

$$\min_{𝒵,ℰ}∥𝒵{∥}_{⊛r}+\lambda_{1}\sum_{v=1}^{n_{3}}∥𝑀⊙{𝒵}^{\left(v\right)}{∥}_{\ell_{q}}^{q}+\lambda_{2}∥ℰ{∥}_{2,p}+\lambda_{3}\sum_{v=1}^{n_{3}}tr({𝒵}^{\left(v\right)}{𝐿}^{\left(v\right)}({𝒵}^{\left(v\right)}{)}^{⊤})\;s.t.\;𝒳=𝒳*𝒵+ℰ,\;{𝒵}_{i,i,k}=0,$$ (22)

where $\forall i,j=1,2,\ldots ,N,\;k=1,2,\ldots ,n_{3}$, $\lambda_{1},\lambda_{2}$, and $\lambda_{3}$ are balance parameters. The flowchart of the 2D-NLRSC is shown in Fig 1. Our method processes the input image data as a third-order tensor, successfully preserving the original spatial structure of images and avoiding structural information loss due to vectorization. The first term employs the $\ell_{r}$-induced tensor nuclear norm to impose a low-rank constraint on the representation tensor, thereby capturing the global subspace structure of the data. The second term applies the $\ell_{q}$-norm to the Hadamard product of the representation tensor and the dissimilarity weighted matrix, promoting element-wise sparsity and forming a block-diagonal structure conducive to clustering. The third term uses the $\ell_{2,p}$-norm to limit the error tensor, boosting column-wise sparsity and model robustness against outliers and noise. The fourth term uses Laplacian regularization to utilize the local geometric structure of the data, ensuring that the representation preserves neighborhood interactions within the intrinsic manifold and so strengthens intra-cluster cohesiveness and inter-cluster separation. .

By solving (22), the optimal representation tensor can be used to construct affinity matrices.The affinity matrix *S* is formulated as

$$S_{ij}=\frac{1}{2}\left(\|𝒵(i,j,:){\|}_{2}^{2}+\|𝒵(j,i,:){\|}_{2}^{2}\right).$$ (23)

Then, spectral clustering or the normalized graph cut algorithm is applied to the affinity matrix *S* to derive the final clustering outcomes.

### 4.2 Optimization

Three auxiliary variables 𝒞, 𝒬, 𝒯 are introduced into our optimization algorithm, which can be expressed as follows

$$\min_{𝒵,𝒞,𝒬,ℰ,𝒯}\|𝒞{\|}_{⊛r}+\lambda_{1}\sum_{v=1}^{n_{3}}\|𝑀⊙{𝒬}^{\left(v\right)}{\|}_{\ell_{q}}^{q}+\lambda_{2}\|ℰ{\|}_{2,p}+\lambda_{3}\sum_{v=1}^{n_{3}}tr\left({𝒯}^{\left(v\right)}{𝐿}^{\left(v\right)}({𝒯}^{\left(v\right)}{)}^{⊤}\right)\;\text{s.t.}\;𝒵=𝒞,\;𝒵=𝒬,\;𝒵=𝒯,\;𝒳=𝒳*𝒵+ℰ.$$ (24)

The augmented Lagrangian function for this problem can be expressed as

$$ℒ(𝒵,𝒞,𝒬,𝒯,ℰ)=\;\|𝒞{\|}_{⊛r}+\lambda_{1}\sum_{v=1}^{n_{3}}{\left\|𝑀⊙{𝒬}^{\left(v\right)}\right\|}_{\ell_{q}}^{q}+\lambda_{2}\|ℰ{\|}_{2,p}\;+\lambda_{3}\sum_{v=1}^{n_{3}}tr\left({𝒯}^{\left(v\right)}{𝐿}^{\left(v\right)}({𝒯}^{\left(v\right)}{)}^{⊤}\right)+\frac{\mu_{1}}{2}({\left\|𝒵-𝒞+\frac{{𝒢}_{1}}{\mu_{1}}\right\|}_{F}^{2}+{\left\|𝒵-𝒬+\frac{{𝒢}_{2}}{\mu_{1}}\right\|}_{F}^{2}\;+{\left\|𝒵-𝒯+\frac{{𝒢}_{3}}{\mu_{1}}\right\|}_{F}^{2})+\frac{\mu_{2}}{2}{\left\|𝒳-𝒳*𝒵-ℰ+\frac{𝒴}{\mu_{2}}\right\|}_{F}^{2}.$$ (25)

1) 𝒞 Subproblem: Fixing all variables except 𝒞, the update for 𝒞 is

$${𝒞}^{*}=\arg\min_{𝒞}\|𝒞{\|}_{⊛r}+\frac{\mu_{1}}{2}{\left\|𝒞-\left(𝒵+\frac{{𝒢}_{1}}{\mu_{1}}\right)\right\|}_{F}^{2}.$$ (26)

**Theorem 2.** Let $𝒜\in {\mathbb{R}}^{n_{1}\times n_{2}\times n_{3}}$ have the *t*-SVD decomposition $𝒜=𝒰*𝒮*{𝒱}^{⊤}$. Consider the $\ell_{r}$-induced tensor nuclear norm optimization problem

$$\underset{𝒳}{\arg\min}\mu \|𝒳{\|}_{⊛r}+\frac{1}{2}\|𝒳-𝒜{\|}_{F}^{2},$$ (27)

the optimal solution is given by

$${𝒳}^{*}=\Gamma_{\mu }[𝒜]=𝒰*ifft(P_{\mu }(\hat{𝒜}))*{𝒱}^{⊤},$$ (28)

where $P_{\mu }(\hat{𝒜})\in {\mathbb{R}}^{n_{1}\times n_{2}\times n_{3}}$ is an *f*-diagonal tensor, whose diagonal elements are generated by the GST algorithm described in (16).

Applying Theorem 2, the optimal solution of (26) is

$${𝒞}^{*}=\Gamma_{\frac{1}{\mu_{1}}}\left(𝒵+\frac{{𝒢}_{1}}{\mu_{1}}\right)=𝒰\cdot ifft\left(P_{\frac{1}{\mu_{1}}}\left(\hat{𝒵}+\frac{{\hat{𝒢}}_{1}}{\mu_{1}}\right)\right)\cdot {𝒱}^{⊤}.$$ (29)

2) 𝒬 Subproblem: The update for 𝒬 is

$${𝒬}^{*}=\arg\min_{𝒬}\lambda_{1}\sum_{v=1}^{n_{3}}{\left\|𝑀⊙{𝒬}^{\left(v\right)}\right\|}_{\ell_{q}}^{q}+\frac{\mu_{1}}{2}{\left\|𝒬-\left(𝒵+\frac{{𝒢}_{2}}{\mu_{1}}\right)\right\|}_{F}^{2}.$$ (30)

Following the GST algorithm described in (16), the closed-form solution is

$${𝒬}_{ij}^{(k{)}^{*}}={𝕋}_{q}^{GST}\left({𝒵}_{ij}^{\left(k\right)}+\frac{{𝒢}_{2_{ij}}^{\left(k\right)}}{\mu_{1}},\varepsilon_{ij}\right),$$ (31)

where $\varepsilon_{ij}=\frac{\lambda_{1}}{\mu_{1}}M_{ij}$.

3) 𝒯 Subproblem: The update for 𝒯 is

$${𝒯}^{(v{)}^{*}}=\arg\min_{{𝒯}^{\left(v\right)}}\lambda_{3}\sum_{v=1}^{n_{3}}tr\left({𝒯}^{\left(v\right)}{𝐿}^{\left(v\right)}({𝒯}^{\left(v\right)}{)}^{⊤}\right)+\frac{\mu_{1}}{2}{\left\|𝒯-\left(𝒵+\frac{{𝒢}_{3}}{\mu_{1}}\right)\right\|}_{F}^{2}.$$ (32)

Setting the partial derivatives with respect to ${𝒯}^{\left(v\right)}$ to zero gives

$$2\lambda_{3}{𝒯}^{\left(v\right)}L^{\left(v\right)}+\mu_{1}\left({𝒯}^{\left(v\right)}-\left({𝒵}^{\left(v\right)}+\frac{{𝒢}_{3}^{\left(v\right)}}{\mu_{1}}\right)\right)=0$$ (33)

The optional solution for ${𝒯}^{\left(v\right)}$ is derived as

$${𝒯}^{(v{)}^{*}}=\left(\mu_{1}{𝒵}^{\left(v\right)}+{𝒢}_{3}^{\left(v\right)}\right){\left(\mu_{1}𝐼+2\lambda_{3}{𝐿}^{\left(v\right)}\right)}^{-1}.$$ (34)

4) ℰ Subproblem: The update rule for ℰ is formulated as

$${ℰ}^{*}=\arg\min_{ℰ}\lambda_{2}\|ℰ{\|}_{2,p}+\frac{\mu_{2}}{2}{\left\|𝒳-𝒳*𝒵-ℰ+\frac{𝒴}{\mu_{2}}\right\|}_{F}^{2}\;\Rightarrow \arg\min_{{ℰ}^{\left(v\right)}}\lambda_{2}\|{ℰ}^{\left(v\right)}{\|}_{2,p}+\frac{\mu_{2}}{2}{\left\|\;{ℰ}^{\left(v\right)}-{𝒲}^{\left(v\right)}\right\|}_{F}^{2},$$ (35)

where ${𝒲}^{\left(v\right)}={𝒳}^{\left(v\right)}-{𝒮}^{\left(v\right)}+\frac{{𝒴}^{\left(v\right)}}{\mu_{2}}$, and ${𝒮}^{\left(v\right)}$ is the *v*-th frontal slice of 𝒳*𝒵.

Let $\beta =\frac{\lambda_{2}}{\mu_{2}}$, ${𝐸}_{:,i}$ be the symbol for the *i*-th column of *E*, and take ${𝑊}_{:,i}$ to mean the *i*-th column of $𝑊=[{𝒲}^{\left(1\right)};\cdots ;{𝒲}^{\left(v\right)}]$. The objective in (35) can be reformulated column-wise as

$$\min_{{𝐸}_{:,i}}\sum_{i=1}^{N}\left\{\frac{1}{2}\|{𝑊}_{:,i}-{𝐸}_{:,i}{\|}_{2}^{2}+\beta \|{𝐸}_{:,i}{\|}_{2}^{p}\right\},$$ (36)

so that each ${𝐸}_{:,i}$ can be solved separately. For a specific ${𝐸}_{:,i}$, the subproblem becomes

$$\min_{{𝐸}_{:,i}}\frac{1}{2}\|{𝑊}_{:,i}-{𝐸}_{:,i}{\|}_{2}^{2}+\beta \|{𝐸}_{:,i}{\|}_{2}^{p}.$$ (37)

Here, ${𝐸}_{:,i}$ can be treated as a special matrix, and a thin SVD can be applied. It follows that ${𝐸}_{:,i}$ has exactly one singular value, given by

$$\sigma ({𝐸}_{:,i})=\sqrt{{𝐸}_{:,i}^{⊤}{𝐸}_{:,i}}=\|{𝐸}_{:,i}{\|}_{2},$$ (38)

where σ(·) represents the singular value of the input vector. Hence, the subproblem is equivalent to

$$\min_{{𝐸}_{:,i}}\frac{1}{2}\|{𝑊}_{:,i}-{𝐸}_{:,i}{\|}_{2}^{2}+\beta \sigma ({𝐸}_{:,i}{)}^{p}.$$ (39)

Now, we introduce Lemma 1.

**Lemma 1.** [31] Consider two complex matrices *A* and $𝐵\in {\mathbb{C}}^{m\times n}$. Let $F:{\mathbb{C}}^{m\times n}\to \mathbb{C}$ be defined as

$$F(𝐴)=f\circ {\vec{\sigma }}_{𝐴}=f(\sigma_{1}(𝐴),\ldots ,\sigma_{K}(𝐴)),$$ (40)

where ${\vec{\sigma }}_{𝐴}$ represents the vector consisting of the non-increasing singular values of *A*. If F(𝐴) is a complex unitarily invariant function (or quasi-norm) and *B* has an SVD formulated as

$$𝐵=𝑈\Sigma_{𝐵}{𝑉}^{⊤},$$ (41)

then the optimal solution ${𝐴}^{*}$ for the minimization problem

$$\min_{𝐴}\left\{\frac{1}{2}\|𝐴-𝐵{\|}_{F}^{2}+F(𝐴)\right\},$$ (42)

has the SVD $U\Sigma_{𝐴}^{*}{𝑉}^{⊤}$, where

$$\Sigma_{𝐴}^{*}=diag({\vec{\sigma }}_{𝐴}^{*}).$$ (43)

and

$${\vec{\sigma }}_{𝐴}^{*}=\arg\min_{\vec{\sigma }}\left\{\frac{1}{2}\|\vec{\sigma }-{\vec{\sigma }}_{𝐵}{\|}_{2}^{2}+f(\vec{\sigma })\right\}.$$ (44)

According to Lemma 1, the solution to (39) is expressed as

$${𝐸}_{:,i}=u_{i}\sigma^{*}({𝐸}_{:,i})v_{i}^{⊤},$$ (45)

where *u*_*i*_ and $v_{i}$ are respectively the left and right singular vectors corresponding to ${𝑊}_{:,i}$, and

$$\sigma^{*}({𝐸}_{:,i})=\arg\min_{x\geq 0}\frac{1}{2}(\sigma ({𝑊}_{:,i})-x{)}^{2}+\beta x^{p},$$ (46)

which can be computed using the GST algorithm in (16).

It is evident that(refer to 7.2 of [32])

$${𝑊}_{:,i}=\frac{{𝑊}_{:,i}}{\|{𝑊}_{:,i}{\|}_{2}}\|{𝑊}_{:,i}{\|}_{2}[1],$$ (47)

denotes a thin SVD of ${𝑊}_{:,i}$, where [1] represents a matrix with 1 as its only element. By replacing

$$u_{i}=\frac{{𝑊}_{:,i}}{\|{𝑊}_{:,i}{\|}_{2}},\sigma ({𝑊}_{:,i})=\|{𝑊}_{:,i}{\|}_{2},$$ (48)

$v_{i}=[1]$ into (45),we can derive ${𝑊}_{:,i}$.

5) 𝒵 Subproblem: The update for 𝒵 is

$${𝒵}^{*}=\arg\min_{𝒵}\frac{\mu_{1}}{2}\left(\|𝒵-{𝒫}_{1}{\|}_{F}^{2}+\|𝒵-{𝒫}_{2}{\|}_{F}^{2}+\|𝒵-{𝒫}_{3}{\|}_{F}^{2}\right)+\frac{\mu_{2}}{2}\|𝒪-𝒳*𝒵{\|}_{F}^{2}.$$ (49)

where ${𝒫}_{1}=𝒞-\frac{{𝒢}_{1}}{\mu_{1}},{𝒫}_{2}=𝒬-\frac{{𝒢}_{2}}{\mu_{1}},{𝒫}_{3}=𝒯-\frac{{𝒢}_{3}}{\mu_{1}},𝒪=𝒳-ℰ+\frac{𝒴}{\mu_{2}}$. Using discrete Fourier transform, the solution is

$${\hat{𝒵}}^{(v{)}^{*}}={\left(3\mu_{1}𝐼+\mu_{2}{\hat{𝒳}}^{(v{)}^{⊤}}{\hat{𝒳}}^{\left(v\right)}\right)}^{-1}\left[\mu_{1}\left({\hat{𝒫}}_{1}^{\left(v\right)}+{\hat{𝒫}}_{2}^{\left(v\right)}+{\hat{𝒫}}_{3}^{\left(v\right)}\right)+\mu_{2}{\hat{𝒳}}^{(v{)}^{⊤}}{\hat{𝒪}}^{\left(v\right)}\right].$$ (50)

Overall, the algorithm is summed up in Algorithm 1.

**Algorithm 1 The algorithm of 2D-NLRSC.**

**Input:** Given data tensor 𝒳, dissimilarity matrix M, and   parameters $\lambda_{1}$, $\lambda_{2}$, and $\lambda_{3}$.

**Output:** Representation tensor ${𝒵}^{*}$.

1: Initialization: ${𝒞}^{\left(0\right)}={𝒬}^{\left(0\right)}={𝒯}^{\left(0\right)}={𝒵}^{\left(0\right)}={ℰ}^{\left(0\right)}={𝒴}^{\left(0\right)}=$

${𝒢}_{1}^{\left(0\right)}={𝒢}_{2}^{\left(0\right)}={𝒢}_{3}^{\left(0\right)}=0\in {\mathbb{R}}^{N\times N\times n_{3}}$, penalty parameter $\mu_{1}^{\left(0\right)}=1e-4$,

$\mu_{2}^{\left(0\right)}=1e-5$, ρ=1.8, $\mu_{\text{max}}=10^{10}$, $\varepsilon =10^{-5}$, and *t* = 0.

2: **while** not converge **do**

3:   Fix the others and update 𝒵 by (50);

4:   Fix the others and update ℰ by (45);

5:   Fix the others and update 𝒞 by (29);

6:   Fix the others and update 𝒬 by (31);

7:   Fix the others and update 𝒯 by (34);

8:   Fix the others and update the Lagrange multipliers ${𝒢}_{1}$, ${𝒢}_{2}$,

${𝒢}_{3}$ and 𝒴 by

emsp;   ${𝒢}_{1}^{\left(t+1\right)}={𝒢}_{1}^{\left(t\right)}+\mu_{1}^{\left(t\right)}({𝒵}^{\left(t+1\right)}-{𝒞}^{\left(t+1\right)})$;

${𝒢}_{2}^{\left(t+1\right)}={𝒢}_{2}^{\left(t\right)}+\mu_{1}^{\left(t\right)}({𝒵}^{\left(t+1\right)}-{𝒬}^{\left(t+1\right)})$;

${𝒢}_{3}^{\left(t+1\right)}={𝒢}_{3}^{\left(t\right)}+\mu_{1}^{\left(t\right)}({𝒵}^{\left(t+1\right)}-{𝒯}^{\left(t+1\right)})$;

${𝒴}^{\left(t+1\right)}={𝒴}^{\left(t\right)}+\mu_{2}^{\left(t\right)}(𝒳-𝒳*{𝒵}^{\left(t+1\right)}-{ℰ}^{\left(t+1\right)})$ ;

9:   Update the parameter $\mu_{1}$ and $\mu_{2}$ by

$\mu_{1}^{\left(t+1\right)}=\min(\rho \mu_{1}^{\left(t\right)},\mu_{\text{max}})$;

$\mu_{2}^{\left(t+1\right)}=\min(\rho \mu_{2}^{\left(t\right)},\mu_{\text{max}})$;

10:   Check the convergence conditions if


$$\max\left\{\|{𝒵}^{\left(t+1\right)}-{𝒞}^{\left(t\right)}{\|}_{\infty },\|{𝒵}^{\left(t+1\right)}-{𝒬}^{\left(t\right)}{\|}_{\infty },\|{𝒵}^{\left(t+1\right)}-{𝒯}^{\left(t\right)}{\|}_{\infty },\;\|{𝒵}^{\left(t+1\right)}-{𝒵}^{\left(t\right)}{\|}_{\infty },\|{𝒞}^{\left(t+1\right)}-{𝒞}^{\left(t\right)}{\|}_{\infty },\|{𝒬}^{\left(t+1\right)}-{𝒬}^{\left(t\right)}{\|}_{\infty },\;\|{𝒯}^{\left(t+1\right)}-{𝒯}^{\left(t\right)}{\|}_{\infty },\|{ℰ}^{\left(t+1\right)}-{ℰ}^{\left(t\right)}{\|}_{\infty },\;\|𝒳-𝒳*{𝒵}^{\left(t+1\right)}-{ℰ}^{\left(t+1\right)}{\|}_{\infty },\right\}<\varepsilon$$

11:   **Output**: Representation tensor ${𝒵}^{*}$.

12:   else, *t* = *t* + 1;

13: **end while**

### 4.3 Complexity analysis

The computational cost of our method primarily arises from the updates of $𝒞\in {\mathbb{R}}^{N\times N\times n_{3}}$ and $𝒵\in {\mathbb{R}}^{N\times N\times n_{3}}$. When updating 𝒞, it is necessary to compute the FFT and inverse FFT of an $N\times N\times n_{3}$ tensor along mode-3, as well as perform the SVD of N×N matrices in the Fourier domain. These operations require $𝒪(2N^{2}n_{3}\log(n_{3})+N^{3}n_{3})$ computations per iteration. For updating 𝒵, the process involves calculating the matrix inverse and the FFT and inverse FFT transformations. The computational complexity of these operations is $𝒪(4N^{2}n_{3}\log(n_{3})+N^{3}n_{3})$. Consequently, the total computational complexity of the method is approximately $𝒪(6T_{1}N^{2}n_{3}\log(n_{3})+2T_{1}N^{3}n_{3})$, where *T*_1_ denotes the number of iterations required to solve (24) using ADMM.

## 5 Experiments

### 5.1 Datasets

Five image datasets are selected for this algorithm, and they are described in Table 2 as follows

1) **ORL**: The ORL dataset comprises 40 distinct subjects, each represented by 10 different images. The experimental settings align with those described in [16]. All 400 images are utilized, and each image is of a dimension of 32×32.
2) **JAFFE**: The JAFFE dataset comprises 213 images of 7 facial expressions from ten Japanese female subjects. Ten participants were selected for this study, each contributing their first 20 images. All 200 images are resized to 23×23. The experimental setup follows the same approach outlined in [16].
3) **CMU-PIE**: The CMU-PIE face dataset comprises 42,368 images of 68 subjects, exhibiting diverse poses, lighting conditions, and facial expressions. A subset of 735 images was created, containing the initial 49 images from each of the first 15 subjects. These images were subsequently resized to 32×32. The specific experiments and settings are in [16].
4) **Yale**: The Yale face dataset consists of 165 images of 15 individuals, each with a size of 100×100. 11 distinct facial images, exhibiting varied expressions and lighting, were provided by each participant. These images were uniformly resized to 25×25. The experimental conditions are aligned with those described in [16].
5) **MNIST**:The MNIST dataset contains 70,000 centered 28×28 images of handwritten digits (0-9). A subset of 1,000 images was created by selecting the first 100 images of each digit. The specific experimental settings are described in [16]. For each value of L∈{3,5,8,10}, the clustering process was repeated 20 times with randomly selected categories. The average of these 20 clustering results was then used for evaluation.
6) **COIL-20**:The COIL-20 dataset contains 1,440 images from 20 different object categories. Each object category has 72 images captured from different angles, and the backgrounds of the objects were removed during the shooting process. These images were processed and downsampled to a size of 25×25 .

**Table 2:** Statistics of the image datasets in the experiments.

Datasets	Objective	Clusters	Images
Yale	Face	15	165
JAFFE	Face	10	200
ORL	Face	40	400
CMU-PIE	Face	15	735
MNIST	Digits	10	1000
COIL-20	Object	20	1440

In our experiment, Accuracy (ACC) and Normalized Mutual Information (NMI) are utilized as performance evaluation metrics. Higher values for both ACC and NMI indicate superior clustering performance.

### 5.2 Compared clustering algorithms

To evaluate the performance of the algorithms proposed in this paper, we selected the state-of-the-art clustering algorithms as benchmarks for our experimental comparisons

- LRR [3] is a clustering algorithm that reveals the latent structure in data by constructing a low-rank representation matrix.
- SSC [2] solves a sparse optimization program to derive the sparse representation of data points from other points.
- LSR [33] leverages data correlation to achieve subspace segmentation through a grouping effect that tends to cluster highly correlated data together, significantly improving segmentation accuracy while ensuring efficiency.
- SC-LRR [34] extends the standard LRR by introducing a predefined weight matrix to analyze the structure of multiple disjoint subspaces, breaking through the restriction of the standard LRR that requires subspaces to be independent.
- TSC [35] achieves clustering of noisy and incompletely observed high-dimensional data into a union of low-dimensional subspaces and outliers by thresholding the correlations between data points to obtain an adjacency matrix.
- S3C [36] learns both the affinity matrix and segmentation results simultaneously through a joint optimization framework, expressing each data point as a structured, sparse linear combination of other data points.
- KSSC [37] extends sparse subspace clustering to nonlinear manifolds via the kernel trick, enhancing clustering performance through nonlinear mappings.
- SSmC [26] integrates the *t*-product operation into the sparse subspace clustering framework, enhancing the model’s ability to capture local features of data through its convolutional structure.
- SCLRSmC [14] introduces a free submodules theory, enabling low-rank representation learning directly in the tensor space and avoiding the inherent loss of structural information associated with traditional vectorization preprocessing.
- CLLRSmC [16] considers the intrinsic manifold structure of data and integrates the two distinct stages of learning a low-rank representation tensor and performing spectral clustering into a unified optimization framework.
- KCLLRSmC [16] combines manifold regularization with kernel methods for manifold clustering, eliminating the need for explicit mapping of data into the feature space. It serves as a nonlinear extension of CLLRSmC.

### 5.3 Experiments results and analysis

The subsequent experiments use ACC and NMI as clustering evaluation metrics. Their precise definitions are provided in [16]. Each experiment is replicated 20 times, and the average results are recorded in Tables 3, 4, 5, and 6. The best results are highlighted in bold. As shown in the tables, the proposed model significantly outperforms other models regarding both ACC and NMI across the ORL, JAFFE, CMU-PIE, Yale, and MNIST datasets.

**Table 3:** Experimental results on ORL and JAFFE datasets. The parameters are set as $\lambda_{1}=0.001$, $\lambda_{2}=0.001$, $\lambda_{3}=0.1$ on ORL; $\lambda_{1}=0.0001$, $\lambda_{2}=0.001$, $\lambda_{3}=0.0001$ on JAFFE.

Dataset	ORL		JAFFE	
Metric	ACC	NMI	ACC	NMI
LRR	0.7800	0.8722	0.8617	0.8793
SCC	0.8525	0.9224	0.8219	0.8576
LSR1	0.7475	0.8721	0.9645	0.9523
LSR2	0.7725	0.8856	0.9738	0.9660
SC-LRR	0.8050	0.9086	0.9760	0.9835
TSC	0.5250	0.7742	0.9089	0.9216
S3C	0.7825	0.9243	0.8318	0.8632
KSSC	0.7600	0.8613	0.8845	0.8729
SSmC	0.7975	0.8862	0.9172	0.9005
SCLRSmC	0.8525	0.9198	0.9900	0.9854
CLLRSmC	0.8825	0.9369	0.9921	0.9886
KCLLRSmC	0.8925	0.9423	0.9964	0.9923
2D-NLRSC	0.8975	0.9477	1.0000	1.0000

**Table 4:** Experimental results on CMU-PIE and Yale datasets. The parameters are set as $\lambda_{1}=0.0001$, $\lambda_{2}=0.001$, $\lambda_{3}=0.0001$ on CMU-PIE; $\lambda_{1}=0.0001$, $\lambda_{2}=0.001$, $\lambda_{3}=0.0001$ on Yale.

Dataset	CMU-PIE		Yale	
Metric	ACC	NMI	ACC	NMI
LRR	0.9190	0.8864	0.6970	0.7091
SCC	0.8471	0.9196	0.6986	0.7006
LSR1	0.9198	0.8910	0.7012	0.6988
LSR2	0.9227	0.9068	0.7093	0.6994
SC-LRR	0.9161	0.9088	0.7215	0.7381
TSC	0.9174	0.9034	0.7020	0.6985
S3C	0.9180	0.8919	0.7292	0.7203
KSSC	0.9208	0.8954	0.7369	0.7311
SSmC	0.9214	0.9056	0.7558	0.7740
SCLRSmC	0.9312	0.9247	0.7776	0.7984
CLLRSmC	0.9582	0.9523	0.8815	0.8919
KCLLRSmC	0.9618	0.9580	0.9242	0.9336
2D-NLRSC	0.9850	0.9744	0.9345	0.9413

**Table 5:** Experimental results on MNIST dataset (*L* = 3 and *L* = 5). The parameters are set as $\lambda_{1}=0.01$, $\lambda_{2}=0.001$, $\lambda_{3}=10$ on MNIST.

Dataset	*L* *= 3*		*L* *= 5*	
Metric	ACC	NMI	ACC	NMI
LRR	0.5313	0.4268	0.4290	0.3714
SCC	0.4961	0.4226	0.3889	0.3492
LSR1	0.4946	0.4251	0.3951	0.3322
LSR2	0.5008	0.4344	0.3932	0.3326
SC-LRR	0.4889	0.4103	0.4254	0.3670
TSC	0.4844	0.4027	0.4491	0.3673
S3C	0.5212	0.4046	0.4418	0.3906
KSSC	0.5634	0.4350	0.4916	0.3372
SSmC	0.6427	0.4305	0.5367	0.4003
SCLRSmC	0.7348	0.5472	0.6061	0.4939
CLLRSmC	0.7520	0.5708	0.6271	0.5263
KCLLRSmC	0.7710	0.6039	0.6611	0.5775
2D-NLRSC	**0.7926**	**0.6398**	**0.6648**	**0.5783**

**Table 6:** Experimental results on MNIST dataset (*L* = 8 and *L* = 10). The parameters are set as $\lambda_{1}=0.01$, $\lambda_{2}=0.001$, $\lambda_{3}=10$ on MNIST.

Dataset	*L* *= 8*		*L* *= 10*	
Metric	ACC	NMI	ACC	NMI
LRR	0.3668	0.3804	0.3421	0.3565
SCC	0.3450	0.3405	0.3633	0.3620
LSR1	0.3198	0.3624	0.3404	0.3418
LSR2	0.3219	0.3638	0.3458	0.3639
SC-LRR	0.3869	0.4095	0.4115	0.3856
TSC	0.4255	0.4251	0.4360	0.4139
S3C	0.4130	0.4245	0.4021	0.4148
KSSC	0.4269	0.3792	0.4220	0.3669
SSmC	0.4884	0.4193	0.4512	0.4155
SCLRSmC	0.5716	0.5353	0.5564	0.5088
CLLRSmC	0.5922	0.5468	0.5698	0.5320
KCLLRSmC	0.6018	**0.5677**	0.5940	0.5416
2D-NLRSC	**0.6350**	0.5406	**0.5992**	**0.5419**

On the ORL dataset, our method outperforms all other models, achieving improvements of 0.50% in ACC and 1.50% in NMI compared to the suboptimal KCLLRSmC.

On the JAFFE dataset, as shown in Table 3, the proposed model achieves a perfect score of 100% for both ACC and NMI. In particular, our model outperforms the suboptimal KCLLRSmC model by 0.36% in ACC and 0.77% in NMI.

Experiments on the CMU-PIE dataset demonstrate that 2D-NLRSC achieves superior performance compared to existing methods, yielding the highest ACC and NMI scores. Specifically, it surpasses the suboptimal method by 2.32% in ACC and 0.54% in NMI.

On the Yale dataset, 2D-NLRSC achieved the highest ACC and NMI scores, surpassing the best-performing suboptimal method by 1.03% and 0.77%, respectively.

On the MNIST dataset, compared to all other methods, the proposed 2D-NLRSC shows significant improvements in ACC and NMI. When the number of categories *L* = 8, the NMI is slightly lower than those of KCLLRSmC and CLLRSmC, but outperforms other algorithms. Compared with KCLLRSmC, when *L* = 3, the ACC and NMI metrics are improved by 2.16% and 3.59%, respectively. Similarly, when *L* = 5 and *L* = 10, the ACC and NMI metrics are improved by {0.37%, 0.52%} and {0.08%, 0.03%}, respectively.

**Fig 2:**
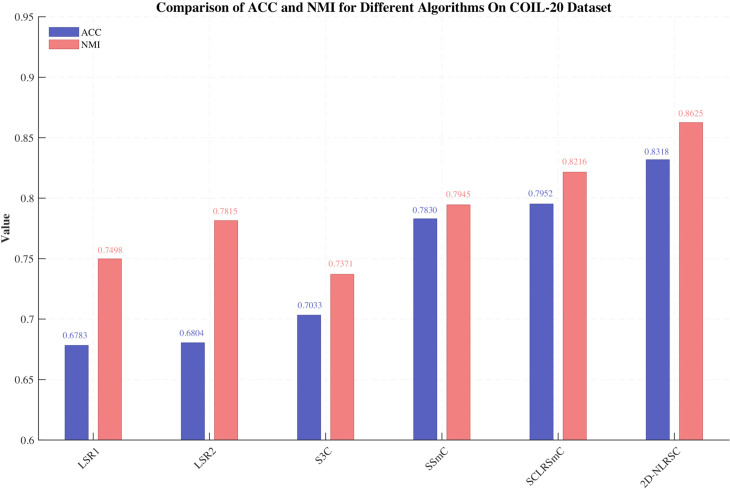
Comparison of ACC and NMI for Different Algorithms On COIL-20 Dataset.

Experimental evaluations on the COIL-20 dataset, detailed in Fig 2, illustrate that the 2D-NLRSC method yields significant advancements in clustering performance, reflected by its elevated ACC and NMI scores.

In summary, the proposed 2D-NLRSC method significantly improves almost all datasets. These results strongly validate that 2D-NLRSC can more effectively capture high-order correlation information in samples through tensor rank approximation and joint sparse regularization based on the $\ell_{q}$-norm and the $\ell_{2,p}$-norm.

### 5.4 Ablation studies

**Fig 3:**
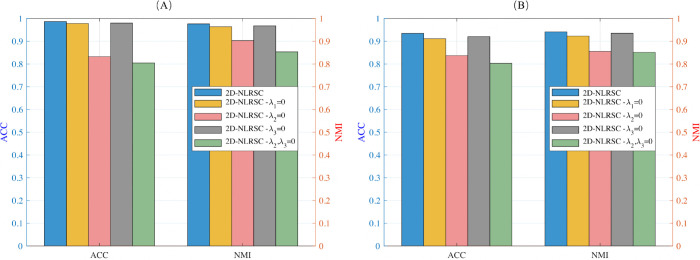
Ablation study of the proposed method: Comparisons on dataset (A) CMU-PIE and dataset (B) Yale.

To validate the role of each regularization term in the model, ablation experiments were conducted. Specifically, partial regularization terms were removed from the model 2D-NLSRC, and parameters were adjusted to achieve the optimal performance of the model. On the CMU-PIE and Yale dataset, three groups of experiments were designed, with each experiment omitting one regularization term to derive three algorithms. Through parameter optimization for each algorithm, the impact of each regularization term on the model’s performance was evaluated. The specific experimental results are shown in Fig 3.

According to Fig 3, the suggested 2D-NLSRC method outperforms all other ablation variants in terms of clustering performance, as measured by ACC and NMI. We observed similar performance deterioration across all incomplete configurations in systematic ablation trials with individual regularization factors removed. On the CMU-PIE dataset, the complete 2D-NLSRC model improves ACC by {0.86%, 15.42%, 0.64%, 18.19%} and NMI by {1.22%, 7.34%, 0.83%, 12.29%} over the ablation versions 2D-NLSRC($\lambda_{1}=0$), 2D-NLSRC($\lambda_{2}=0$), 2D-NLSRC($\lambda_{3}=0$), and 2D-NLSRC($\lambda_{1},\lambda_{2}=0$). The Yale dataset shows performance increases of {2.36%, 9.76%, 1.45%, 13.18%} in ACC and {1.84%, 8.59%, 0.61%, 9.02%} in NMI.

These empirical findings give significant support for the efficacy of multi-component collaboration in our approach. The performance loss found when $\lambda_{1}=0$ emphasizes the necessity of the $\ell_{q}$-norm regularization that works in conjunction with the inconsistency matrix *M*. This combination applies differentiated constraints to sample pairs from various submodules, promoting the construction of a distinct block-diagonal structure in the representation tensor. The performance drop at $\lambda_{2}=0$ confirms that the $\ell_{2,p}$-norm introduces crucial column-wise sparse constraints, effectively eliminating interference from redundant information and outliers. The performance degradation observed when $\lambda_{3}=0$ demonstrates that the Laplacian regularization term effectively preserves the local geometric structure of the data, and its absence leads to disrupted neighborhood relationships that are crucial for maintaining clustering coherence The significant performance degradation in the dual-ablated scenario 2D-NLSRC ($\lambda_{1},\lambda_{2}=0$) highlights the complimentary nature of these two regularization techniques, which jointly contribute to the improved clustering accuracy of 2D-NLSRC on complex datasets.

### 5.5 Sensitivity analysis

To comprehensively evaluate the robustness and stability of the proposed algorithm, we conduct a sensitivity analysis focusing on two key aspects: the parameter K in K-Nearest Neighbors (KNN) and the initialization of core variables (𝒵,𝒞,𝒬,𝒯).

#### 5.5.1 Sensitivity analysis of KNN parameter K.

**Fig 4:**
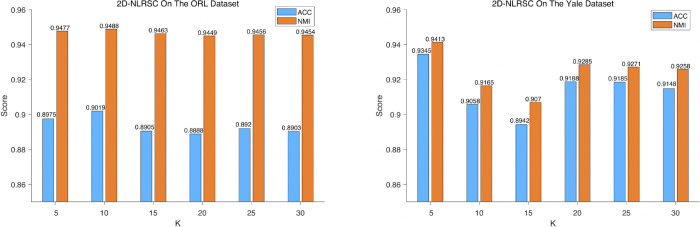
The performance of 2D-NLRSC on the ORL and Yale datasets under different K values.

We set the range of K from 5 to 30 and conduct experiments on both ORL and Yale datasets, with the specific results shown in Fig 4. The experimental data indicates that: on the ORL dataset, when K is in the range [5, 30], the fluctuation range of the algorithm’s accuracy ACC is controlled within 1.5%, and the fluctuation of normalized mutual information NMI does not exceed 1%; on the Yale dataset, the ACC fluctuation range is within 4%, and the NMI fluctuation does not exceed 3.6% . This result fully demonstrates the algorithm’s robustness to changes in K . Further analysis reveals that the relationship between K and algorithm performance is not linear. Within the tested range, the experimental results indicate that setting K=5 yields superior performance. Therefore, K is set to 5 for the subsequent experiments.

#### 5.5.2 Sensitivity analysis of initialization of core variables (𝒵,𝒞,𝒬,𝒯).

To comprehensively investigate the impact of the initialization of core variables (𝒵,𝒞,𝒬,𝒯) on the 2D-NLRSC algorithm, we design multiple sets of comparative experiments: we perform 5 repeated experiments for zero initialization and three types of random Gaussian initializations with different variances (𝒩(0,0.1), 𝒩(0,0.5), 𝒩(0,1)), and the final results are summarized in Table 7 after taking the average.On the ORL dataset, both ACC and NMI of zero initialization are the highest among all initialization methods; on the Yale dataset, its ACC and NMI are also significantly better than various random initializations, indicating that zero initialization can provide a better initial starting point for the algorithm, enabling the model to converge to a solution of higher quality. As the variance of the Gaussian distribution increases from 0.1 to 1, the ACC on the ORL dataset decreases from 0.8974 to 0.8785, and the NMI decreases from 0.9474 to 0.9408; on the Yale dataset, the ACC decreases from 0.9318 to 0.9273, and the NMI decreases from 0.9408 to 0.9348. It can be seen that the performance of random initialization shows a downward trend as the variance increases, and the larger the variance, the more obvious the performance degradation, indicating that the algorithm has a certain sensitivity to the variance change of random initialization.

**Table 7:** Performance comparison of 2D-NLRSC under different initializations on ORL and Yale datasets.

Initialization Method	ORL Dataset			Yale Dataset		
	ACC	NMI	Iterations	ACC	NMI	Iterations
Zero Initialization	**0.8975**	**0.9477**	**41**	**0.9345**	**0.9413**	**36**
Random (𝒩(0,0.1))	0.8974	0.9474	41	0.9318	0.9408	36
Random (𝒩(0,0.5))	0.8916	0.9429	41	0.9270	0.9338	36
Random (𝒩(0,1))	0.8785	0.9408	41	0.9273	0.9348	36

For both the ORL dataset (with 41 iterations for all) and the Yale dataset (with 36 iterations for all), the number of convergence iterations of different initialization methods is completely consistent. This indicates that although initialization affects the quality of the solution, it has no significant interference with the convergence speed of the algorithm, reflecting the stability of the algorithm in terms of convergence efficiency. Therefore, based on the comprehensive experimental evidence that zero initialization consistently achieves superior performance on both datasets without impairing convergence speed, it is adopted as the initialization method of choice in this paper.

### 5.6 Parameter sensitivity

In our method, $\lambda_{1}$, $\lambda_{2}$, and $\lambda_{3}$ serve as balancing parameters, whereas *r*, *p*, and *q* denote hyperparameters. We systematically investigated their impact on clustering performance (quantified by ACC and NMI) across five benchmark datasets. Through exhaustive grid search, optimal parameter configurations were empirically determined for each dataset, as presented in Tables 3–6.

**Fig 5:**
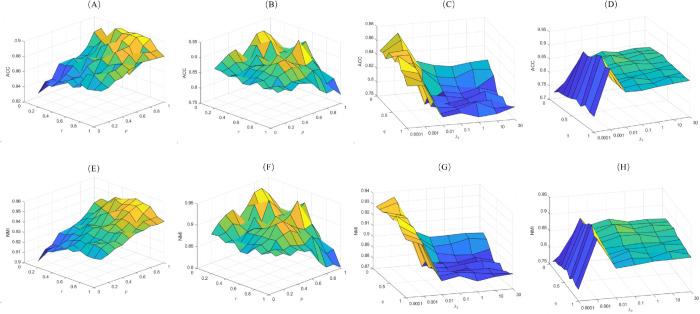
ACC and NMI of the 2D-NLRSC method on ORL and Yale datasets: Dependence on parameters r,p,q and $\lambda_{2}$ ((A),(E): ORL-r,p; (B),(F): Yale-r,p; (C),(G): ORL-$q,\lambda_{2}$; (D),(H): Yale-$q,\lambda_{2}$).

**Fig 6:**
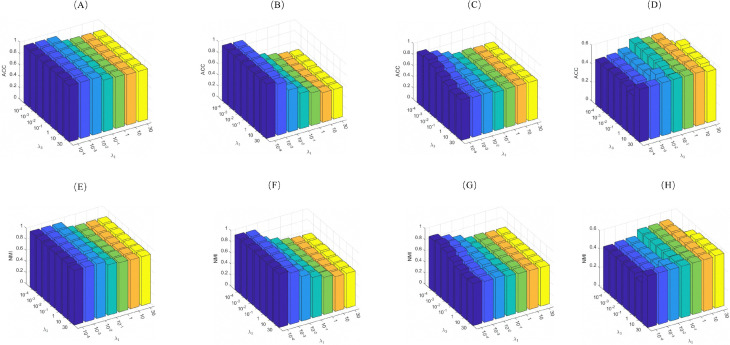
ACC and NMI of the 2D-NLRSC method on JAFFE, CMU-PIE, Yale and MNIST (L = 10) datasets: Dependence on parameters $\lambda_{1}$ and $\lambda_{3}$ ((A),(E): JAFFE; (B),(F): CMU-PIE; (C),(G): Yale; (D),(H): MNIST (L = 10)).

For the ORL and Yale datasets, we fixed all parameters except *r* and *p*, which were varied within {0.1,0.2,0.3,0.4,0.5,0.6,0.7,0.8,0.9,1} to isolate their effects. To analyze the joint influence of *q* and $\lambda_{2}$ on Yale and ORL datasets, we set $\lambda_{2}$ $\in \{10^{-4},10^{-3},10^{-2},10^{-1},1,10,30\}$ and *q* ∈{0.1,0.2,0.3,0.4,0.5,0.6,0.7,0.8,0.9,1} while keeping the other variables constant. For the JAFFE, CMU-PIE, Yale and MNIST (*L* = 10) datasets, $\lambda_{2}$ is fixed at 0.001, and $\lambda_{1}$, $\lambda_{3}$ were varied within $\left\{10^{-4},10^{-3},10^{-2},10^{-1},1,10,30\right\}$ to evaluate their regulatory effects. Visualizations of these parameter analyses are provided in Figs 5 and 6.

As shown in Fig 5, the value of *r* does not always correlate positively with clustering performance. On the ORL and Yale datasets, when *r* approaches 1, the clustering effect significantly declines, whereas setting *r* around 0.5 yields the best results. For parameter *p*, a threshold effect is evident: when *p*<0.7, clustering performance is constrained. As *p* increases, the algorithm performance improves gradually, however, when *p* = 1, the $\ell_{2,p}$-norm degenerates into the $\ell_{2,1}$-norm, which weakens sparsity constraints and degrades ACC. Based on the experimental results, the optimal range for *p* is [0.7,1). Parameter *q* exhibits a relatively stable influence, with stable and superior clustering performance observed when its value is within [0.4,0.9]. To ensure generalization ability and clustering performance of the model across different datasets, this paper selects q={0.9,0.9,0.5,0.9,0.5,0.6}, p={0.9,0.9,0.9,0.7,0.9,0.7}, r={0.5,0.5,0.5,0.6,0.5,0.6} for the ORL, JAFFE, CMU-PIE, Yale, MNIST and COIL-20 datasets, respectively.

As shown in Fig 6, after parameter tuning, the ACC and NMI of our algorithm exhibit similar trends across different datasets. Notably, when $\lambda_{1}>0.1$, the clustering performance deteriorates significantly. Similarly, as shown in Fig 5, on the Yale dataset, the performance also degrades when $\lambda_{2}>0.1$. Based on empirical results, we recommend setting $\lambda_{1}\leq 0.1$, $\lambda_{2}\leq 0.1$, and $\lambda_{3}\leq 1$ (as $\lambda_{3}$ has a relatively stable impact).

Additionally, the running times of the 2D-NLRSC on different datasets are provided. As clearly shown in the Table 8, our algorithm exhibits significantly shorter running times than the CLIRSmc and KCLIRSmc algorithms on the ORL, JAFFE, and CMU-PIE datasets. Furthermore, our algorithm achieves higher ACC and NMI values compared to the KCLIRSmc algorithm. Overall, our algorithm demonstrates an acceptable time complexity.

**Table 8:** Running time (sec) comparison on different datasets.

Dataset/Methods	LRR	SSC	LSR1	SC-LRR	TSC	S3C	KSSC	SSmC	SCLRSmc	CLIRSmc	KCLIRSmc	2D-NLRSC
ORL	11.22	12.84	10.58	18.36	25.70	20.45	21.05	37.69	66.17	98.14	106.36	80.36
JAFFE	3.52	1.63	1.43	5.32	5.61	5.77	9.10	12.24	14.59	18.43	20.30	8.43
CMU-PIE	22.59	15.49	21.13	25.17	55.30	72.86	110.32	158.60	223.48	278.78	301.55	245.60

## 6 Convergence analysis

**Fig 7:**
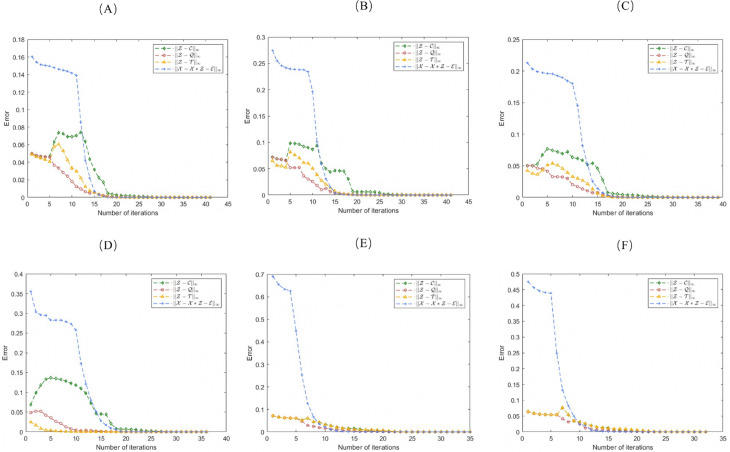
Error convergence curves of 2D-NLRSC on different datasets ((A): ORL; (B): JAFFE; (C): CMU-PIE; (D): Yale; (E): MNIST (*L* = 3); (F): MNIST (*L* = 5)).

The convergence of the 2D-NLRSC is examined from two distinct perspectives in this section. First, a rigorous theoretical analysis is provided to establish the convergence properties of the proposed method. Second, the convergence behavior is demonstrated through plots of various variables, as shown in Fig 7.

**Lemma 2.** [31] Suppose $F:{\mathbb{R}}^{n_{1}\times n_{2}}\to \mathbb{R}$ is portrayed as F(X)=f∘σ(X), where $X\in {\mathbb{R}}^{n_{1}\times n_{2}}$ has the SVD $X=U\mathrm{diag}(\sigma_{1},\ldots ,\sigma_{r})V^{⊤}$, $r=\min(n_{1},n_{2})$, and *f* is differentiable. The gradient of *F*(*X*) at *X* is

$$\frac{\partial F(X)}{\partial X}=U\mathrm{diag}(\theta )V^{⊤},$$ (51)

where $\theta =\frac{\partial f(y)}{\partial y}{|}_{y=\sigma (X{)}^{*}}$.

**Lemma 3.** [38] If the matrix *H* is positive definite, then

$$(x,Hx)\geq a\|x{\|}^{2},\;\text{for any vector }x,$$ (52)

where (·,·) represents the inner product, and *a* stands for the smallest eigenvalue of *H*.

**Lemma 4.** [38] In the finite-dimensional Euclidean space, every bounded sequence of vectors has a subsequence that converges.

**Lemma 5.** [39] Consider the nonconvex optimization problem with $\ell_{q}$ regularization

$$\min_{𝐱\in {\mathbb{R}}^{n}}\left\{F(𝐱):=f(𝐱)+\lambda \|𝐱{\|}_{\ell_{q}}^{q}\right\},$$ (53)

where λ>0, q∈(0,1), and $\|𝐱{\|}_{q}:=(\sum_{i=1}^{n}|x_{i}{|}^{q}{)}^{1/q}$. Assume the function *f* satisfies the following conditions: *f* has *L*_*f*_-Lipschitz continuous gradient, i.e.,

$$\|\nabla f(𝐱)-\nabla f(𝐲){\|}_{2}\leq L_{f}\|𝐱-𝐲{\|}_{2},\;\forall 𝐱,𝐲\in {\mathbb{R}}^{n},$$ (54)

and *f* is bounded below on ${\mathbb{R}}^{n}$.

Then the following properties hold

(i) If ${𝐱}^{*}$ is a local minimizer of (53), then it satisfies the first-order stationary condition
  $${𝐗}^{*}\nabla f({𝐱}^{*})+\lambda q|{𝐱}^{*}{|}^{q}=0,$$ (55)
  where ${𝐗}^{*}=\text{diag}({𝐱}^{*})$ and $|{𝐱}^{*}{|}^{q}=(|x_{1}^{*}{|}^{q},\cdots ,|x_{n}^{*}{|}^{q}{)}^{T}$.
(ii) Let ${𝐱}^{*}$ be a stationary point with $F({𝐱}^{*})\leq F({𝐱}^{0})+\epsilon_{1}$ for some initial point ${𝐱}^{0}\in {\mathbb{R}}^{n}$ and small $\epsilon_{1}>0$. Define $f_{\text{min}}=\inf_{𝐱\in {\mathbb{R}}^{n}}f(𝐱)$ and let $\text{supp}({𝐱}^{*})=\{i:x_{i}^{*}\neq 0\}$. Then each nonzero component satisfies
  $$|x_{i}^{*}|\geq {\left(\frac{\lambda q}{\sqrt{2L_{f}\left[F({𝐱}^{0})+\epsilon_{1}-f_{\text{min}}\right]}}\right)}^{\frac{1}{1-q}},\;\forall i\in \text{supp}({𝐱}^{*}).$$ (56)

**Lemma 6.** ${{𝒢}_{1}}^{\left(t+1\right)}$ is bounded.

*Proof:* In order to achieve the minimization of 𝒞 at the (t+1)-th step as illustrated in equation (26), the optimal solution ${𝒞}^{\left(t+1\right)}$ must satisfy the following condition

$$\frac{\partial {\left\|{𝒞}^{\left(t+1\right)}\right\|}_{⊛r}}{\partial {𝒞}^{\left(t+1\right)}}+\mu_{1}^{\left(t\right)}({𝒞}^{\left(t+1\right)}-{𝒵}^{\left(t\right)}-\frac{{𝒢}_{1}^{\left(t\right)}}{\mu_{1}^{\left(t\right)}})=0,$$ (57)

where r∈(0,1]. The expression $(|\eta {|}^{r}{)}^{\prime }=\frac{r\;1pt\eta }{|\eta {|}^{2-r}}$ has a singularity near η=0. To circumvent this, we propose an approximation for η with 0<ϵ≪1

$$\frac{\partial |\eta {|}^{r}}{\partial \eta }\approx \frac{r\;1pt\eta }{\max\{\epsilon^{2-r},|\eta {|}^{2-r}\}}.$$ (58)

Let ${𝒞}^{\left(t\right)}$ be expressed as ${𝒰}^{\left(t\right)}\mathrm{diag}\left(\sigma_{j}({𝒞}^{\left(t\right)})\right){𝒱}^{(t{)}^{⊤}}$. From Definition 4 and Lemma 2, it can be deduced that

$$\frac{\partial {\left\|{\hat{𝒞}}^{\left(t\right)}\right\|}_{⊛r}}{\partial {\hat{𝒞}}^{\left(t\right)}}={\hat{𝒰}}^{\left(t\right)}\mathrm{diag}\left(\frac{r\sigma_{j}({\hat{𝒞}}^{\left(t\right)})}{\max\{\epsilon^{2-r},|\sigma_{j}({\hat{𝒞}}^{\left(t\right)}){|}^{2-r}\}}\right){\hat{𝒱}}^{(t{)}^{⊤}}.$$ (59)

Then we have

$$\frac{r\sigma_{j}({\hat{𝒞}}^{\left(t\right)})}{\max\{\epsilon^{2-r},|\sigma_{j}({\hat{𝒞}}^{\left(t\right)}){|}^{2-r}\}}\leq \frac{r}{\epsilon^{1-r}}\Rightarrow \sum_{t=1}^{V}{\left\|\frac{\partial {\left\|{\hat{𝒞}}^{\left(t\right)}\right\|}_{⊛r}}{\partial {\hat{𝒞}}^{\left(t\right)}}\right\|}_{F}^{2}\leq \frac{Vr^{2}}{\epsilon^{2(1-r)}}.$$ (60)

Therefore, it can be seen that $\frac{\partial {\left\|\hat{𝒞}\right\|}_{⊛r}}{\partial \hat{𝒞}}$ is bounded. We can denote ${\tilde{F}}_{V}=\frac{1}{\sqrt{V}}F_{V}$, where $F_{n_{3}}$ is the DFT matrix of size V×V, and $F_{n_{3}}^{⊤}$ is its conjugate transpose. Given that $𝒞=\hat{𝒞}\;\;\times_{3}\;\;{\tilde{F}}_{n_{3}}$, the following result is derived using the chain rule of matrix calculus

$$\frac{\partial \|𝒞{\|}_{⊛r}}{\partial 𝒞}=\frac{\partial \|𝒞{\|}_{⊛r}}{\partial \hat{𝒞}}\times_{3}{\tilde{F}}_{V}^{⊤},$$ (61)

is bounded.

From the relations ${{𝒢}_{1}}^{\left(t+1\right)}={{𝒢}_{1}}^{\left(t\right)}+\mu_{1}^{\left(t\right)}({𝒵}^{\left(t+1\right)}-{𝒞}^{\left(t+1\right)})$ and $\frac{\partial {\left\|{𝒞}^{\left(t+1\right)}\right\|}_{⊛r}}{\partial {𝒞}^{\left(t+1\right)}}={{𝒢}_{1}}^{\left(t+1\right)}$, it follows that ${{𝒢}_{1}}^{\left(t+1\right)}$ is bounded. ◻

**Lemma 7.** ${{𝒢}_{2}}^{\left(t+1\right)}$ is bounded.

*Proof:* To prove that ${{𝒢}_{2}}^{\left(t+1\right)}$ is bounded, consider the minimization of 𝒬 at the (t+1)-th step as illustrated in (30). The optimal solution ${𝒬}^{\left(t+1\right)}$ must satisfy

$$\lambda_{1}\frac{\partial {\left\|M⊙{𝒬}^{(v{)}^{\left(t+1\right)}}\right\|}_{\ell_{q}}^{q}}{\partial {𝒬}^{(v{)}^{\left(t+1\right)}}}+\mu_{1}^{\left(t\right)}({𝒬}^{(v{)}^{\left(t+1\right)}}-{𝒵}^{(v{)}^{\left(t\right)}}-\frac{{𝒢}_{2}^{(v{)}^{\left(t\right)}}}{\mu_{1}^{\left(t\right)}})=0.$$ (62)

From the update ${𝒢}_{2}^{\left(t+1\right)}={𝒢}_{2}^{\left(t\right)}+\mu_{1}^{\left(t\right)}({𝒵}^{\left(t+1\right)}-{𝒬}^{\left(t+1\right)})$, it follows that

$$\lambda_{1}\frac{\partial {\left\|M⊙{𝒬}^{(v{)}^{\left(t+1\right)}}\right\|}_{\ell_{q}}^{q}}{\partial {𝒬}^{(v{)}^{\left(t+1\right)}}}={𝒢}_{2}^{(v{)}^{\left(t+1\right)}}.$$ (63)

The boundedness of the $\ell_{q}$ norm with power *q* is bounded [40] implies the boundedness of ${𝒢}_{2}^{\left(t+1\right)}$. ◻

**Lemma 8.** ${{𝒢}_{3}}^{\left(t+1\right)}$ is bounded.

*Proof:* To achieve the minimization of 𝒯 at the (t+1)-th step as illustrated in (32), the optimal solution ${𝒯}^{\left(t+1\right)}$ requires to satisfy the following

$${𝒯}^{(v{)}^{\left(t+1\right)}}(\mu_{1}𝐼+2\lambda_{3}L^{(v{)}^{\left(t+1\right)}})-\mu_{1}{𝒵}^{(v{)}^{\left(t+1\right)}}-{𝒢}_{3}^{(v{)}^{\left(t\right)}}=0,$$ (64)

due to the ${𝒢}_{3}$ update by ${𝒢}_{3}^{\left(t+1\right)}={𝒢}_{3}^{\left(t\right)}+\mu_{1}^{\left(t\right)}({𝒵}^{\left(t+1\right)}-{𝒯}^{\left(t+1\right)})$, then we can obtain

$${𝒢}_{3}^{(v{)}^{\left(t+1\right)}}\}{𝒢}_{3}^{(v{)}^{\left(t+1\right)}}=2\lambda_{3}{𝒯}^{(v{)}^{\left(t+1\right)}}L^{(v{)}^{\left(t+1\right)}}.$$ (65)

Based on the proof above, auxiliary variable ${𝒞}^{\left(t+1\right)}$ is bounded, which implies that ${𝒵}^{\left(t+1\right)}$ and its associated auxiliary variable ${𝒯}^{\left(t+1\right)}$ are also bounded. According to the triangle inequality, it can be obtained that

$${\left\|{𝒢}_{3}^{(v{)}^{\left(t+1\right)}}\right\|}_{F}={\left\|2\lambda_{3}{𝒯}^{(v{)}^{\left(t+1\right)}}L^{(v{)}^{\left(t+1\right)}}\right\|}_{F}\;\leq 2\lambda_{3}{\left\|{𝒯}^{(v{)}^{\left(t+1\right)}}\right\|}_{F}{\left\|L^{(v{)}^{\left(t+1\right)}}\right\|}_{F}<+\infty .$$ (66)

Clearly, ${{𝒢}_{3}}^{\left(t+1\right)}$ is bounded. ◻

**Lemma 9.** $Y^{\left(t+1\right)}$ is bounded.

*Proof:* Consider the =of ℰ at the (t+1)-th step as illustrated in (35). The optimal solution ${ℰ}^{\left(t+1\right)}$ must satisfy

$$\frac{\lambda_{2}}{\mu_{2}}\frac{\partial \|{ℰ}^{\left(t+1\right)}{\|}_{2,p}}{\partial {ℰ}^{\left(t+1\right)}}+{ℰ}^{\left(t+1\right)}-\left(𝒳-𝒳*{𝒵}^{\left(t+1\right)}+\frac{{𝒴}^{\left(t\right)}}{\mu_{2}^{\left(t\right)}}\right)=0.$$ (67)

Given 0<ϵ≪1, the partial derivative of ${\left\|{ℰ}_{:,i}^{\left(v\right)}\right\|}_{2,p}$ with respect to ${ℰ}_{:,i}^{\left(v\right)}$ is expressed as follows

$$\frac{\partial {\left\|{ℰ}_{:,i}^{\left(v\right)}\right\|}_{2,p}}{\partial {ℰ}_{:,i}^{\left(v\right)}}=\left\{ \begin{array}{cc} \frac{p\sigma ({ℰ}_{:,i}^{\left(v\right)})}{\max\{\epsilon^{2-p},|\sigma ({ℰ}_{:,i}^{\left(v\right)}){|}^{2-p}\}}\frac{{ℰ}_{:,i}^{\left(v\right)}}{{\left\|{ℰ}_{:,i}^{\left(v\right)}\right\|}_{2}}, & {ℰ}_{:,i}^{\left(v\right)}\neq 0,\\ \frac{p}{1-p}\frac{p\sigma ({ℰ}_{:,i}^{\left(v\right)})}{\max\{\epsilon^{2-p},|\sigma ({ℰ}_{:,i}^{\left(v\right)}){|}^{2-p}\}}{\left\|{ℰ}_{:,i}^{\left(v\right)}\right\|}_{2}, & {ℰ}_{:,i}^{\left(v\right)}=0. \end{array}\right.$$ (68)

Thus, the following inequality holds

$${\left\|\frac{\partial {\left\|{ℰ}_{:,i}^{\left(v\right)}\right\|}_{2,p}}{\partial {ℰ}_{:,i}^{\left(v\right)}}\right\|}_{\ell_{2}}\leq \frac{p}{1-p}.$$ (69)

From the update ${𝒴}^{\left(t+1\right)}={𝒴}^{\left(t\right)}+\mu_{2}^{\left(t\right)}(𝒳-𝒳*{𝒵}^{\left(t+1\right)}-{ℰ}^{\left(t+1\right)})$, this can be rewritten as

$$\lambda_{2}\frac{\partial \|{ℰ}^{\left(t+1\right)}{\|}_{2,p}}{\partial {ℰ}^{\left(t+1\right)}}-{𝒴}^{\left(t+1\right)}=0.$$ (70)

Thus, ${𝒴}^{\left(t+1\right)}$ is bounded. ◻

**Theorem 3.** Let $\{{𝒫}^{\left(t\right)}=({𝒵}^{\left(t\right)},{ℰ}^{\left(t\right)},{𝒬}^{\left(t\right)},{𝒞}^{\left(t\right)},{𝒯}^{\left(t\right)},{𝒴}^{\left(t\right)},{𝒢}_{1}^{\left(t\right)},{𝒢}_{2}^{\left(t\right)},{𝒢}_{3}^{\left(t\right)}){\}}_{t=1}^{\infty }$ be the sequence generated by Algorithm 1. Then the sequence $\{{𝒫}^{\left(t\right)}{\}}_{t=1}^{\infty }$ satisfies the following two principles:

1. $\{{𝒫}^{\left(t\right)}{\}}_{t=1}^{\infty }$ is bounded.
2. Any accumulation point of $\{{𝒫}^{\left(t\right)}{\}}_{t=1}^{\infty }$ is a KKT point of (25).

*Proof: 1) Proof of the first part of Theorem 3:*

Given the following update rules

$${𝒢}_{1}^{\left(t\right)}={𝒢}_{1}^{\left(t-1\right)}+\mu_{1}^{\left(t-1\right)}({𝒵}^{\left(t\right)}-{𝒞}^{\left(t\right)}),\;{𝒢}_{2}^{\left(t\right)}={𝒢}_{2}^{\left(t-1\right)}+\mu_{1}^{\left(t-1\right)}({𝒵}^{\left(t\right)}-{𝒬}^{\left(t\right)}),\;{𝒢}_{3}^{\left(t\right)}={𝒢}_{3}^{\left(t-1\right)}+\mu_{1}^{\left(t-1\right)}({𝒵}^{\left(t\right)}-{𝒯}^{\left(t\right)}),\;{𝒴}^{\left(t\right)}={𝒴}^{\left(t-1\right)}+\mu_{2}^{\left(t-1\right)}(𝒳-𝒳*{𝒵}^{\left(t\right)}-{ℰ}^{\left(t\right)}).$$ (71)

It can be deduced that

$${ℒ}_{\mu_{1}^{\left(t\right)},\mu_{2}^{\left(t\right)}}({𝒵}^{\left(t+1\right)},{ℰ}^{\left(t+1\right)},{𝒬}^{\left(t+1\right)},{𝒞}^{\left(t+1\right)},{𝒯}^{\left(t+1\right)},{𝒴}^{\left(t\right)},{𝒢}_{1}^{\left(t\right)},{𝒢}_{2}^{\left(t\right)},{𝒢}_{3}^{\left(t\right)})\;\leq {ℒ}_{\mu_{1}^{\left(t\right)},\mu_{2}^{\left(t\right)}}({𝒵}^{\left(t\right)},{ℰ}^{\left(t\right)},{𝒬}^{\left(t\right)},{𝒞}^{\left(t\right)},{𝒯}^{\left(t\right)},{𝒴}^{\left(t\right)},{𝒢}_{1}^{\left(t\right)},{𝒢}_{2}^{\left(t\right)},{𝒢}_{3}^{\left(t\right)})\;={ℒ}_{\mu_{1}^{\left(t-1\right)},\mu_{2}^{\left(t-1\right)}}({𝒵}^{\left(t\right)},{ℰ}^{\left(t\right)},{𝒬}^{\left(t\right)},{𝒞}^{\left(t\right)},{𝒯}^{\left(t\right)},{𝒴}^{\left(t-1\right)},{𝒢}_{1}^{\left(t-1\right)},{𝒢}_{2}^{\left(t-1\right)},{𝒢}_{3}^{\left(t-1\right)})\;+\sum_{i=1}^{3}\left(\frac{\mu_{1}^{\left(t\right)}+\mu_{1}^{\left(t-1\right)}}{2(\mu_{1}^{\left(t-1\right)}{)}^{2}}{\left\|{𝒢}_{i}^{\left(t\right)}-{𝒢}_{i}^{\left(t-1\right)}\right\|}_{F}^{2}+\frac{{\left\|{𝒢}_{i}^{\left(t\right)}\right\|}_{F}^{2}}{2\mu_{1}^{\left(t\right)}}-\frac{{\left\|{𝒢}_{i}^{\left(t-1\right)}\right\|}_{F}^{2}}{2\mu_{1}^{\left(t-1\right)}}\right)\;+\frac{\mu_{2}^{\left(t\right)}+\mu_{2}^{\left(t-1\right)}}{2(\mu_{2}^{\left(t-1\right)}{)}^{2}}{\left\|{𝒴}^{\left(t\right)}-{𝒴}^{\left(t-1\right)}\right\|}_{F}^{2}+\frac{{\left\|{𝒴}^{\left(t\right)}\right\|}_{F}^{2}}{2\mu_{2}^{\left(t\right)}}-\frac{{\left\|{𝒴}^{\left(t-1\right)}\right\|}_{F}^{2}}{2\mu_{2}^{\left(t-1\right)}}.$$ (72)

Summing both sides of Eq (72) from *t* = 1 to *k*, we have

$${ℒ}_{\mu_{1}^{\left(k\right)},\mu_{2}^{\left(k\right)}}({𝒵}^{\left(k+1\right)},{ℰ}^{\left(k+1\right)},{𝒬}^{\left(k+1\right)},{𝒞}^{\left(k+1\right)},{𝒯}^{\left(k+1\right)},{𝒴}^{\left(k\right)},{𝒢}_{1}^{\left(k\right)},{𝒢}_{2}^{\left(k\right)},{𝒢}_{3}^{\left(k\right)})\;\leq {ℒ}_{\mu_{1}^{\left(0\right)},\mu_{2}^{\left(0\right)}}({𝒵}^{\left(1\right)},{ℰ}^{\left(1\right)},{𝒬}^{\left(1\right)},{𝒞}^{\left(1\right)},{𝒯}^{\left(1\right)},{𝒴}^{\left(0\right)},{𝒢}_{1}^{\left(0\right)},{𝒢}_{2}^{\left(0\right)},{𝒢}_{3}^{\left(0\right)})\;+\sum_{i=1}^{3}\left(\sum_{t=1}^{k}\left(\frac{\mu_{1}^{\left(t\right)}+\mu_{1}^{\left(t-1\right)}}{2(\mu_{1}^{\left(t-1\right)}{)}^{2}}{\left\|{𝒢}_{i}^{\left(t\right)}-{𝒢}_{i}^{\left(t-1\right)}\right\|}_{F}^{2}\right)+\frac{{\left\|{𝒢}_{i}^{\left(k\right)}\right\|}_{F}^{2}}{2\mu_{1}^{\left(k\right)}}-\frac{{\left\|{𝒢}_{i}^{\left(0\right)}\right\|}_{F}^{2}}{2\mu_{1}^{\left(0\right)}}\right)\;+\sum_{t=1}^{k}\left(\frac{\mu_{2}^{\left(t\right)}+\mu_{2}^{\left(t-1\right)}}{2(\mu_{2}^{\left(t-1\right)}{)}^{2}}{\left\|{𝒴}^{\left(t\right)}-{𝒴}^{\left(t-1\right)}\right\|}_{F}^{2}\right)+\frac{{\left\|{𝒴}^{\left(k\right)}\right\|}_{F}^{2}}{2\mu_{2}^{\left(k\right)}}-\frac{{\left\|{𝒴}^{\left(0\right)}\right\|}_{F}^{2}}{2\mu_{2}^{\left(0\right)}}.$$ (73)

Since the sequences $\left\{{{𝒢}_{i}}^{\left(h\right)}\right\}$ and $\left\{{𝒴}^{\left(h\right)}\right\}$ are bounded, and

$$\sum_{t=1}^{+\infty }\frac{\mu_{1}^{\left(t\right)}+\mu_{1}^{\left(t-1\right)}}{2{\left(\mu_{1}^{\left(t-1\right)}\right)}^{2}}=\frac{\rho +1}{2\mu_{1}^{\left(0\right)}}\sum_{t=1}^{+\infty }\frac{1}{\rho^{\left(t-1\right)}}=\frac{\rho \left(\rho +1\right)}{2\mu_{1}^{\left(0\right)}\left(\rho -1\right)}<+\infty ,\;\sum_{t=1}^{+\infty }\frac{\mu_{2}^{\left(t\right)}+\mu_{2}^{\left(t-1\right)}}{2{\left(\mu_{2}^{\left(t-1\right)}\right)}^{2}}=\frac{\rho +1}{2\mu_{2}^{\left(0\right)}}\sum_{t=1}^{+\infty }\frac{1}{\rho^{\left(t-1\right)}}=\frac{\rho \left(\rho +1\right)}{2\mu_{2}^{\left(0\right)}\left(\rho -1\right)}<+\infty ,\;$$ (74)

It can be found that the right of (73) is finite. Thus ${ℒ}_{\mu_{1}^{\left(k\right)},\mu_{2}^{\left(k\right)}}({𝒵}^{\left(k+1\right)},{ℰ}^{\left(k+1\right)},{𝒬}^{\left(k+1\right)},{𝒞}^{\left(k+1\right)},{𝒯}^{\left(k+1\right)},{𝒴}^{\left(k\right)},{𝒢}_{1}^{\left(k\right)},{𝒢}_{2}^{\left(k\right)},{𝒢}_{3}^{\left(k\right)})$ is bounded. We observed

$${ℒ}_{\mu_{1}^{\left(k\right)},\mu_{2}^{\left(k\right)}}({𝒵}^{\left(k+1\right)},{ℰ}^{\left(k+1\right)},{𝒬}^{\left(k+1\right)},{𝒞}^{\left(k+1\right)},{𝒯}^{\left(k+1\right)},{𝒴}^{\left(k\right)},{𝒢}_{1}^{\left(k\right)},{𝒢}_{2}^{\left(k\right)},{𝒢}_{3}^{\left(k\right)})\;=\|{𝒞}^{\left(k+1\right)}{\|}_{⊛r}+\lambda_{1}\sum_{v=1}^{n_{3}}{\left\|𝑀⊙{𝒬}^{\left(v)(k+1\right)}\right\|}_{\ell_{q}}^{q}+\lambda_{2}\|{ℰ}^{\left(k+1\right)}{\|}_{2,p}\;+\lambda_{3}\sum_{v=1}^{n_{3}}tr({𝒯}^{\left(v)(k+1\right)}{𝐿}^{\left(v)(k+1\right)}\left(({𝒯}^{\left(v\right)}{)}^{\left(k+1\right)}{)}^{⊤}\right)\;+\frac{\mu_{1}^{\left(k\right)}}{2}({\left\|{𝒵}^{\left(k+1\right)}-{𝒞}^{\left(k+1\right)}+\frac{{𝒢}_{1}^{\left(k\right)}}{\mu_{1}^{\left(k\right)}}\right\|}_{F}^{2}+{\left\|{𝒵}^{\left(k+1\right)}-{𝒬}^{\left(k+1\right)}+\frac{{𝒢}_{2}^{\left(k\right)}}{\mu_{1}^{\left(k\right)}}\right\|}_{F}^{2}\;+{\left\|{𝒵}^{\left(k+1\right)}-{𝒯}^{\left(k+1\right)}+\frac{{𝒢}_{3}^{\left(k\right)}}{\mu_{1}^{\left(k\right)}}\right\|}_{F}^{2})+\frac{\mu_{2}^{\left(k\right)}}{2}{\left\|𝒳-𝒳*{𝒵}^{\left(k+1\right)}-{ℰ}^{\left(k+1\right)}+\frac{{𝒴}^{\left(k\right)}}{\mu_{2}^{\left(k\right)}}\right\|}_{F}^{2}.$$ (75)

According to Lemma 2, we can find that the right-hand side of (75) is bounded. Since the right of (75) is nonnegative, every term is also bounded. The boundedness of ${\left\|{𝒞}^{\left(k+1\right)}\right\|}_{⊛r}$ means that all singular values of ${𝒞}^{\left(k+1\right)}$ are bounded. Consequently, this ensures that ${\left\|{𝒞}^{\left(k+1\right)}\right\|}_{F}^{2}$ (the sum of the squares of the singular values) is also bounded. Therefore, the sequence $\left\{{𝒞}^{\left(t\right)}\right\}$ is bounded.

According to the Lemma 3,

$$\lambda_{\min}(L)\|({𝒯}^{\left(v\right)}{)}^{\left(k+1\right)}{\|}^{2}\leq \sum_{v=1}^{n_{3}}tr({𝒯}^{\left(v)(k+1\right)}{𝐿}^{\left(v)(k+1\right)}\left(({𝒯}^{\left(v\right)}{)}^{\left(k+1\right)}{)}^{⊤}\right),$$ (76)

where $\lambda_{\min}(L)$ is the smallest positive eigenvalue of the positive definite matrix *L*. Considering the expression $\sum_{v=1}^{n_{3}}tr({𝒯}^{\left(v)(k+1\right)}{𝐿}^{\left(v)(k+1\right)}\left(({𝒯}^{\left(v\right)}{)}^{\left(k+1\right)}{)}^{⊤}\right)$ is bounded, we can further deduce that $\left\{{𝒯}^{\left(t\right)}\right\}$ is bounded.

Given that $\|{ℰ}^{\left(k+1\right)}{\|}_{2,p}\leq M$ for all k≥0 with constant *M*>0 and p∈(0,1], we prove the sequence $\left\{{ℰ}^{\left(t\right)}\right\}$ is bounded. For ${ℰ}^{\left(k+1\right)}\in {\mathbb{R}}^{D\times N\times V}$, the $\ell_{2,p}$- norm is defined as

$$\|{ℰ}^{\left(k+1\right)}{\|}_{2,p}=\|\text{unfold}({ℰ}^{\left(k+1\right)}){\|}_{2,p},$$ (77)

where $H^{\left(k+1\right)}=\text{unfold}({ℰ}^{\left(k+1\right)})\in {\mathbb{R}}^{(DV)\times N}$ satisfies

$$\|H^{\left(k+1\right)}{\|}_{2,p}=\sum_{j=1}^{N}{\left(\sum_{i=1}^{DV}(H_{ij}^{\left(k+1\right)}{)}^{2}\right)}^{\frac{p}{2}}\leq M.$$ (78)

Define $s_{j}^{\left(k+1\right)}=\sum_{i=1}^{DV}(H_{ij}^{\left(k+1\right)}{)}^{2}$ for each column *j*. Then $\sum_{j=1}^{N}(s_{j}^{\left(k+1\right)}{)}^{\frac{p}{2}}\leq M$. As $(s_{j}^{\left(k+1\right)}{)}^{\frac{p}{2}}\geq 0$ and *p* > 0, for all *j*,

$$(s_{j}^{\left(k+1\right)}{)}^{\frac{p}{2}}\leq M⟹s_{j}^{\left(k+1\right)}\leq M^{\frac{2}{p}}.$$ (79)

Since unfold preserves element values, $s_{j}^{\left(k+1\right)}$ corresponds to the sum of squares over all entries of ${ℰ}^{\left(k+1\right)}$ with fixed second index *i*_2_ = *j*,

$$s_{j}^{\left(k+1\right)}=\sum_{i_{1}=1}^{n_{1}}\sum_{i_{3}=1}^{n_{3}}({ℰ}_{i_{1},j,i_{3}}^{\left(k+1\right)}{)}^{2}.$$ (80)

Thus, for any entry ${ℰ}_{i_{1},i_{2},i_{3}}^{\left(k+1\right)}$,

$$({ℰ}_{i_{1},i_{2},i_{3}}^{\left(k+1\right)}{)}^{2}\leq s_{i_{2}}^{\left(k+1\right)}\leq M^{\frac{2}{p}}⟹|{ℰ}_{i_{1},i_{2},i_{3}}^{\left(k+1\right)}|\leq M^{\frac{1}{p}}.$$ (81)

Set $C=M^{\frac{1}{p}}$. Then $|{ℰ}_{i_{1},i_{2},i_{3}}^{\left(k+1\right)}|\leq C$ for all $i_{1},i_{2},i_{3}$. Hence, $\left\{{ℰ}^{\left(t\right)}\right\}$ is bounded.

Similarly, the boundedness of ${\left\|𝑀⊙{𝒬}^{(v{)}^{\left(k+1\right)}}\right\|}_{\ell_{q}}^{q}$ implies that the sequences $\left\{{𝒬}^{\left(k+1\right)}\right\}$ is also bounded. We have

$${𝒵}^{\left(t+1\right)}={𝒞}^{\left(t+1\right)}+\frac{{𝒢}_{1}^{\left(t+1\right)}-{𝒢}_{1}^{\left(t\right)}}{\mu_{1}^{\left(t\right)}},$$ (82)

because ${𝒞}^{\left(t\right)}$ and ${𝒢}_{1}^{\left(t\right)}$ are bounded, we deduce that $\left\{{𝒵}^{\left(t\right)}\right\}$ is also bounded.

In conclusion, it can be proven that $\{{𝒫}^{\left(t\right)}{\}}_{t=1}^{\infty }$ is bounded.

*2) Proof of the second part of Theorem 3:*

Above we have proved that the sequence $\{{𝒫}^{\left(t\right)}{\}}_{t=1}^{\infty }$ generated by Algorithm 1 is ensured to be bounded. According to Lemma 4, which asserts that any bounded sequence in ${\mathbb{R}}^{n}$ possesses a convergent subsequence, it follows that ${𝒫}^{\left(t\right)}$ is bound to have at least a single point of accumulation. Let one of these points be denoted as as ${𝒫}^{*}=\{{𝒵}^{*},{ℰ}^{*},{𝒬}^{*},{𝒞}^{*},{𝒯}^{*},{𝒴}^{*},{𝒢}_{1}^{*},{𝒢}_{2}^{*},{𝒢}_{3}^{*}\}$. Suppose, for the sake of generality, that $\lim_{t\to \infty }{𝒫}^{\left(t\right)}={𝒫}^{*}$.

Based on the update rule regarding ${𝒢}_{1}$, it can be concluded that ${𝒢}_{1}^{\left(t+1\right)}={𝒢}_{1}^{\left(t\right)}+\mu_{1}^{\left(t\right)}({𝒵}^{\left(t+1\right)}-{𝒞}^{\left(t+1\right)})$. Then, by taking the limit of both sides of this equation

$$\lim_{t\to +\infty }\left({𝒵}^{\left(t+1\right)}-{𝒞}^{\left(t+1\right)}\right)=\lim_{t\to +\infty }\left(\frac{{{𝒢}_{1}}^{\left(t+1\right)}-{{𝒢}_{1}}^{\left(t\right)}}{\mu_{1}^{\left(t\right)}}\right)=0,$$ (83)

we obtain ${𝒞}^{*}={𝒵}^{*}$.

Similarly, we obtain ${𝒬}^{*}={𝒯}^{*}={𝒵}^{*}$. Based on the update rule for ${𝒴}^{\left(t\right)}$, it is evident that

$$\lim_{t\to +\infty }\left(\frac{{𝒴}^{\left(t+1\right)}-{𝒴}^{\left(t\right)}}{\mu_{2}^{\left(t\right)}}\right)=\lim_{t\to +\infty }\left(𝒳-𝒳*{𝒵}^{\left(t+1\right)}-{ℰ}^{\left(t+1\right)}\right)=0,$$ (84)

then we can obtain ${ℰ}^{*}=𝒳-𝒳*{𝒵}^{*}.$

Considering the ℰ-subproblem, it is observed that

$$\lambda_{2}\frac{\partial \|{ℰ}^{\left(t+1\right)}{\|}_{2,p}}{\partial {ℰ}^{\left(t+1\right)}}-{𝒴}^{\left(t+1\right)}=0⟹{𝒴}^{*}=\lambda_{2}\frac{\partial \|{ℰ}^{*}{\|}_{2,p}}{\partial {ℰ}^{*}}.$$ (85)

Considering the 𝒞-subproblem, it can be obtained that

$$\frac{\partial {\left\|{𝒞}^{\left(t+1\right)}\right\|}_{⊛r}}{\partial {𝒞}^{\left(t+1\right)}}=G_{1}^{\left(t+1\right)}⟹G_{1}^{*}=\frac{\partial {\left\|{𝒞}^{*}\right\|}_{⊛r}}{\partial {𝒞}^{*}}.$$ (86)

Considering the 𝒬-subproblem, it can be obtained that

$$\lambda_{1}\frac{\partial {\left\|M⊙Q^{(v{)}^{\left(t+1\right)}}\right\|}_{\ell_{q}}^{q}}{\partial Q^{(v{)}^{\left(t+1\right)}}}={𝒢}_{2}^{(v{)}^{\left(t+1\right)}}.$$ (87)

It can be seen that

$${𝒢}_{2}^{*}=\lambda_{1}\sum_{v=1}^{n_{3}}\frac{\partial {\left\|M⊙Q^{(v{)}^{*}}\right\|}_{\ell_{q}}^{q}}{\partial Q^{(v{)}^{*}}}.$$ (88)

Considering the 𝒯-subproblem, we obtain

$$2\lambda_{3}{𝒯}^{(v{)}^{\left(t+1\right)}}{𝐿}^{(v{)}^{\left(t+1\right)}}+\mu_{1}^{\left(t\right)}\left({𝒯}^{(v{)}^{\left(t+1\right)}}-{𝒵}^{(v{)}^{\left(t+1\right)}}\right)-{𝒢}_{3}^{(v{)}^{\left(t\right)}}=0,$$ (89)

and it implies that

$${𝒢}_{3}^{(v{)}^{\left(*\right)}}=2\lambda_{3}{𝒯}^{(v{)}^{\left(*\right)}}L^{(v{)}^{\left(*\right)}}.$$ (90)

Therefore, ${𝒫}^{*}$ satisfies the following KKT conditions

$${𝒞}^{*}={𝒬}^{*}={𝒯}^{*}={𝒵}^{*},\;{𝒴}^{*}=\lambda_{2}\frac{\partial \|{ℰ}^{*}{\|}_{2,p}}{\partial {ℰ}^{*}},G_{1}^{*}=\frac{\partial {\left\|{𝒞}^{*}\right\|}_{⊛r}}{\partial {𝒞}^{*}},\;{ℰ}^{*}=𝒳-𝒳*{𝒵}^{*},\;{𝒢}_{3}^{(v{)}^{\left(*\right)}}=2\lambda_{3}{𝒯}^{(v{)}^{\left(*\right)}}L^{(v{)}^{\left(*\right)}},\;{𝒢}_{2}^{*}=\lambda_{1}\sum_{k=1}^{V}\frac{\partial {\left\|M⊙Q^{(v{)}^{*}}\right\|}_{\ell_{q}}^{q}}{\partial Q^{(v{)}^{*}}}.$$ (91)

The KKT conditions presented below can be applied to determine the termination criteria for Algorithm 1

$$\max\left\{\|{𝒵}^{\left(t+1\right)}-{𝒞}^{\left(t\right)}{\|}_{\infty },\|{𝒵}^{\left(t+1\right)}-{𝒬}^{\left(t\right)}{\|}_{\infty },\|{𝒵}^{\left(t+1\right)}-{𝒯}^{\left(t\right)}{\|}_{\infty },\;\|{𝒵}^{\left(t+1\right)}-{𝒵}^{\left(t\right)}{\|}_{\infty },\|{𝒞}^{\left(t+1\right)}-{𝒞}^{\left(t\right)}{\|}_{\infty },\|{𝒬}^{\left(t+1\right)}-{𝒬}^{\left(t\right)}{\|}_{\infty },\;\|{𝒯}^{\left(t+1\right)}-{𝒯}^{\left(t\right)}{\|}_{\infty },\|{ℰ}^{\left(t+1\right)}-{ℰ}^{\left(t\right)}{\|}_{\infty },\;\|𝒳-𝒳*{𝒵}^{\left(t+1\right)}-{ℰ}^{\left(t+1\right)}{\|}_{\infty },\right\}<\varepsilon$$ (92)

where ε>0 stands for a predefined tolerance. As has been mentioned previously, the sequence complies with the KKT conditions of the Lagrange function (25). ◻

**Theorem 4.** In our algorithm, the sequences $\left\{{𝒵}^{\left(t\right)}\right\}$, $\left\{{𝒞}^{\left(t\right)}\right\}$, $\left\{{𝒬}^{\left(t\right)}\right\}$, $\left\{{𝒯}^{\left(t\right)}\right\}$, and $\left\{{ℰ}^{\left(t\right)}\right\}$ are Cauchy sequences and converge to their critical points.

*Proof of Theorem 4:* We first prove that $\left\{{𝒞}^{\left(t\right)}\right\}$ is a Cauchy sequence. From the update rule ${𝒢}_{1}^{\left(t\right)}={𝒢}_{1}^{\left(t-1\right)}+\mu_{1}^{\left(t-1\right)}({𝒵}^{\left(t\right)}-{𝒞}^{\left(t\right)})$, we can infer the following result

$${\left\|{𝒞}^{\left(t+1\right)}-{𝒞}^{\left(t\right)}\right\|}_{F}={\left\|{𝒞}^{\left(t+1\right)}-\left({𝒵}^{\left(t\right)}+\frac{{𝒢}_{1}^{\left(t\right)}}{\mu_{1}^{\left(t\right)}}\right)+\frac{{𝒢}_{1}^{\left(t\right)}}{\mu_{1}^{\left(t\right)}}+\frac{{𝒢}_{1}^{\left(t\right)}-{𝒢}_{1}^{\left(t-1\right)}}{\mu_{1}^{\left(t-1\right)}}\right\|}_{F}\;\leq {\left\|{𝒞}^{\left(t+1\right)}-{ℱ}^{\left(t\right)}\right\|}_{F}+{\left\|\frac{{𝒢}_{1}^{\left(t\right)}}{\mu_{1}^{\left(t\right)}}+\frac{{𝒢}_{1}^{\left(t\right)}-{𝒢}_{1}^{\left(t-1\right)}}{\mu_{1}^{\left(t-1\right)}}\right\|}_{F},$$ (93)

where ${ℱ}^{\left(t\right)}={𝒵}^{\left(t\right)}+\frac{{𝒢}_{1}^{\left(t\right)}}{\mu_{1}^{\left(t\right)}}$.

Furthermore, this leads to the relation

$${\left\|{𝒞}^{\left(t+1\right)}-{ℱ}^{\left(t\right)}\right\|}_{F}^{2}=\frac{1}{n_{3}}\sum_{v=1}^{n_{3}}{\left\|{\left({\hat{𝒞}}^{\left(v\right)}\right)}^{\left(t+1\right)}-{\left({\hat{ℱ}}^{\left(v\right)}\right)}^{\left(t\right)}\right\|}_{F}^{2}\;=\frac{1}{n_{3}}\sum_{v=1}^{n_{3}}{\left\|{\left({\hat{U}}^{\left(v\right)}\right)}^{\left(t\right)}\left({\left({\hat{S}}^{\left(v\right)}\right)}^{\left(t\right)}-{\left({\hat{\Sigma }}^{\left(v\right)}\right)}^{\left(t\right)}\right){\left({\hat{V}}^{(v{)}^{⊤}}\right)}^{\left(t\right)}\right\|}_{F}^{2}\;=\frac{1}{n_{3}}\sum_{v=1}^{n_{3}}\sum_{l=1}^{N}{\left\|{\left({\hat{S}}_{l,l}^{\left(v\right)}\right)}^{\left(t\right)}-{\left({\hat{\Sigma }}_{l,l}^{\left(v\right)}\right)}^{\left(t\right)}\right\|}_{F}^{2},$$ (94)

where the first equality follows from $\|𝒞{\|}_{F}^{2}=\frac{1}{n_{3}}\|\mathrm{bdiag}(\hat{𝒞}){\|}_{F}^{2}$, the second from Theorem 2, and the third from the unitary invariance of the Frobenius norm.

Recall the subproblem

$${\left({\hat{S}}_{l,l}^{\left(v\right)}\right)}^{\left(t\right)}=\underset{\sigma >0}{\arg\min}\;\frac{\mu_{1}^{\left(t\right)}}{2}{\left(\sigma -{\left({\hat{\Sigma }}_{l,l}^{\left(v\right)}\right)}^{\left(t\right)}\right)}^{2}+\sigma^{r}.$$ (95)

The KKT conditions yield

$$\left\{ \begin{array}{c} \mu_{1}^{\left(t\right)}\left({\left({\hat{S}}_{l,l}^{\left(v\right)}\right)}^{\left(t\right)}-{\left({\hat{\Sigma }}_{l,l}^{\left(v\right)}\right)}^{\left(t\right)}\right)+r{\left({\left({\hat{S}}_{l,l}^{\left(v\right)}\right)}^{\left(t\right)}\right)}^{r-1}-\gamma =0,\\ \gamma \cdot {\left({\hat{S}}_{l,l}^{\left(v\right)}\right)}^{\left(t\right)}=0,\\ {\left({\hat{S}}_{l,l}^{\left(v\right)}\right)}^{\left(t\right)}\geq 0,\\ \gamma \geq 0, \end{array}\right.$$ (96)

where γ is the Lagrange multiplier.

If $\gamma =({\hat{S}}_{l,l}^{\left(v\right)}{)}^{\left(t\right)}=0$, it follow sthat

$${\left({\left({\hat{S}}_{l,l}^{\left(v\right)}\right)}^{\left(t\right)}-{\left({\hat{\Sigma }}_{l,l}^{\left(v\right)}\right)}^{\left(t\right)}\right)}^{2}={\left({\left({\hat{\Sigma }}_{l,l}^{\left(v\right)}\right)}^{\left(t\right)}\right)}^{2}=0\leq \frac{\epsilon^{2}}{{\left(\mu_{1}^{\left(t\right)}\right)}^{2}}.$$ (97)

If γ=0 and $({\hat{S}}_{l,l}^{\left(v\right)}{)}^{\left(t\right)}\neq 0$, it can be concluded that

$${\left({\left({\hat{S}}_{l,l}^{\left(v\right)}\right)}^{\left(t\right)}-{\left({\hat{\Sigma }}_{l,l}^{\left(v\right)}\right)}^{\left(t\right)}\right)}^{2}={\left(\frac{r{\left({\left({\hat{S}}_{l,l}^{\left(v\right)}\right)}^{\left(t\right)}\right)}^{r-1}}{\mu_{1}^{\left(t\right)}}\right)}^{2}\leq \frac{\epsilon^{2}}{{\left(\mu_{1}^{\left(t\right)}\right)}^{2}}.$$ (98)

If γ≠0 and $({\hat{S}}_{l,l}^{\left(v\right)}{)}^{\left(t\right)}=0$, this leads to the conclusion that

$${\left({\left({\hat{S}}_{l,l}^{\left(v\right)}\right)}^{\left(t\right)}-{\left({\hat{\Sigma }}_{l,l}^{\left(v\right)}\right)}^{\left(t\right)}\right)}^{2}={\left({\left({\hat{\Sigma }}_{l,l}^{\left(v\right)}\right)}^{\left(t\right)}\right)}^{2}\leq \frac{\epsilon^{2}}{{\left(\mu_{1}^{\left(t\right)}\right)}^{2}}.$$ (99)

Thus, in all cases, $(({\hat{S}}_{l,l}^{\left(v\right)}{)}^{\left(t\right)}-({\hat{\Sigma }}_{l,l}^{\left(v\right)}{)}^{\left(t\right)}{)}^{2}\leq \frac{\epsilon^{2}}{(\mu_{1}^{\left(t\right)}{)}^{2}}$. Substituting into (94), we obtain the upper bound

$${\left\|{𝒞}^{\left(t+1\right)}-{ℱ}^{\left(t\right)}\right\|}_{F}^{2}\leq \frac{1}{n_{3}}\sum_{v=1}^{n_{3}}\sum_{l=1}^{N}\frac{\epsilon^{2}}{{\left(\mu_{1}^{\left(t\right)}\right)}^{2}}=\frac{N\epsilon^{2}}{{\left(\mu_{1}^{\left(t\right)}\right)}^{2}}.$$ (100)

Therefore,

$${\left\|{𝒞}^{\left(t+1\right)}-{𝒞}^{\left(t\right)}\right\|}_{F}\leq \frac{\sqrt{N}\epsilon }{\mu_{1}^{\left(t\right)}}+\frac{1}{\mu_{1}^{\left(t\right)}}{\left\|{𝒢}_{1}^{\left(t\right)}\right\|}_{F}+\frac{1}{\mu_{1}^{\left(t-1\right)}}{\left\|{𝒢}_{1}^{\left(t\right)}\right\|}_{F}+\frac{1}{\mu_{1}^{\left(t-1\right)}}{\left\|{𝒢}_{1}^{\left(t-1\right)}\right\|}_{F}.$$ (101)

Since $\left\{{𝒢}_{1}^{\left(t\right)}\right\}$ is bounded by *M*_1_ and $\mu_{1}^{\left(t+1\right)}=\rho \mu_{1}^{\left(t\right)}$ with ρ=1.8 and $\mu_{1}^{\left(0\right)}=10^{-4}$, it can therefore be concluded that

$${\left\|{𝒞}^{\left(m\right)}-{𝒞}^{\left(n\right)}\right\|}_{F}\leq \sum_{t=n}^{m-1}{\left\|{𝒞}^{\left(t+1\right)}-{𝒞}^{\left(t\right)}\right\|}_{F}\leq \sum_{t=n}^{m-1}\left(\frac{\sqrt{N}\epsilon }{\rho^{t}\mu_{1}^{\left(0\right)}}+\frac{M_{1}}{\rho^{t}\mu_{1}^{\left(0\right)}}+\frac{2M_{1}}{\rho^{t-1}\mu_{1}^{\left(0\right)}}\right).$$ (102)

The right-hand side is a convergent geometric series, so $\left\{{𝒞}^{\left(t\right)}\right\}$ is a Cauchy sequence.

Next, we prove that $\left\{{𝒵}^{\left(t\right)}\right\}$ is a Cauchy sequence. Using the update ${𝒢}_{1}^{\left(t+1\right)}={𝒢}_{1}^{\left(t\right)}+\mu_{1}^{\left(t\right)}({𝒵}^{\left(t+1\right)}-{𝒞}^{\left(t+1\right)})$, we have

$${\left\|{𝒵}^{\left(t+1\right)}-{𝒵}^{\left(t\right)}\right\|}_{F}={\left\|{𝒞}^{\left(t+1\right)}+\frac{{𝒢}_{1}^{\left(t+1\right)}-{𝒢}_{1}^{\left(t\right)}}{\mu_{1}^{\left(t\right)}}-{𝒞}^{\left(t\right)}-\frac{{𝒢}_{1}^{\left(t\right)}-{𝒢}_{1}^{\left(t-1\right)}}{\mu_{1}^{\left(t-1\right)}}\right\|}_{F}\;\leq {\left\|{𝒞}^{\left(t+1\right)}-{𝒞}^{\left(t\right)}\right\|}_{F}+\frac{1}{\mu_{1}^{\left(t\right)}}{\left\|{𝒢}_{1}^{\left(t+1\right)}\right\|}_{F}+\frac{1}{\mu_{1}^{\left(t\right)}}{\left\|{𝒢}_{1}^{\left(t\right)}\right\|}_{F}\;\;+\frac{1}{\mu_{1}^{\left(t-1\right)}}{\left\|{𝒢}_{1}^{\left(t\right)}\right\|}_{F}+\frac{1}{\mu_{1}^{\left(t-1\right)}}{\left\|{𝒢}_{1}^{\left(t-1\right)}\right\|}_{F}.$$ (103)

Since $\left\{{𝒢}_{1}^{\left(t\right)}\right\}$ is bounded and $\left\{{𝒞}^{\left(t\right)}\right\}$ is a Cauchy sequence, it follows that

$${\left\|{𝒵}^{\left(m\right)}-{𝒵}^{\left(n\right)}\right\|}_{F}\leq \sum_{t=n}^{m-1}{\left\|{𝒵}^{\left(t+1\right)}-{𝒵}^{\left(t\right)}\right\|}_{F}\;\leq \sum_{t=n}^{m-1}\left(\frac{\sqrt{N}\epsilon }{\rho^{t}\mu_{1}^{\left(0\right)}}+\frac{3M_{1}}{\rho^{t}\mu_{1}^{\left(0\right)}}+\frac{4M_{1}}{\rho^{t-1}\mu_{1}^{\left(0\right)}}\right).$$ (104)

Hence, $\left\{{𝒵}^{\left(t\right)}\right\}$ is a Cauchy sequence.

Subsequently, we provide a formal proof that $\left\{{ℰ}^{\left(t\right)}\right\}$ is a Cauchy sequence. Similar to the proof of Lemma 9, we derive the following inequality.

$$\|{𝒴}^{\left(t+1\right)}{\|}_{F}\leq M_{2}$$ (105)

Here, *M*_2_ is its upper bound. From the update ${𝒴}^{\left(t+1\right)}={𝒴}^{\left(t\right)}+\mu_{2}^{\left(t\right)}(𝒳-𝒳*{𝒵}^{\left(t+1\right)}-{ℰ}^{\left(t+1\right)})$, it implies

$${\left\|{ℰ}^{\left(t+1\right)}-𝒳+𝒳*{𝒵}^{\left(t+1\right)}-\frac{{𝒴}^{\left(t\right)}}{\mu_{2}^{\left(t\right)}}\right\|}_{F}\leq \frac{M_{2}}{\mu_{2}^{\left(t\right)}}.$$ (106)

Then,

$${\left\|{ℰ}^{\left(t+1\right)}-{ℰ}^{\left(t\right)}\right\|}_{F}={\left\|{ℰ}^{\left(t+1\right)}-𝒳+𝒳*{𝒵}^{\left(t+1\right)}-\frac{{𝒴}^{\left(t\right)}}{\mu_{2}^{\left(t\right)}}+𝒳-𝒳*{𝒵}^{\left(t+1\right)}+\frac{{𝒴}^{\left(t\right)}}{\mu_{2}^{\left(t\right)}}-{ℰ}^{\left(t\right)}\right\|}_{F}\;\leq {\left\|{ℰ}^{\left(t+1\right)}-𝒳+𝒳*{𝒵}^{\left(t+1\right)}-\frac{{𝒴}^{\left(t\right)}}{\mu_{2}^{\left(t\right)}}\right\|}_{F}+{\left\|𝒳-𝒳*{𝒵}^{\left(t+1\right)}+\frac{{𝒴}^{\left(t\right)}}{\mu_{2}^{\left(t\right)}}-{ℰ}^{\left(t\right)}\right\|}_{F}\;\leq \frac{M_{2}}{\mu_{2}^{\left(t\right)}}+{\left\|𝒳*{𝒵}^{\left(t+1\right)}-𝒳*{𝒵}^{\left(t\right)}\right\|}_{F}+\frac{1}{\mu_{2}^{\left(t\right)}}{\left\|{𝒴}^{\left(t\right)}\right\|}_{F}+\frac{1}{\mu_{2}^{\left(t-1\right)}}{\left\|{𝒴}^{\left(t-1\right)}-{𝒴}^{\left(t\right)}\right\|}_{F}.$$ (107)

Utilizing the linearity property of the *t*-product, we can rewrite the expression as ${\left\|𝒳*{𝒵}^{\left(t+1\right)}-𝒳*{𝒵}^{\left(t\right)}\right\|}_{F}$ = ${\left\|𝒳*\left({𝒵}^{\left(t+1\right)}-{𝒵}^{\left(t\right)}\right)\right\|}_{F}$. Within the framework of tensor *t*-product, the norm typically satisfies the following inequality

$$\|𝒳*𝒜{\|}_{F}\leq \|𝒳{\|}_{2}\cdot \|𝒜{\|}_{F},$$ (108)

where $\|𝒳{\|}_{2}$ denotes the spectral norm of tensor 𝒳. Because 𝒳 is a fixed input tensor and its norm is bounded, then

$${\left\|𝒳*\left({𝒵}^{\left(t+1\right)}-{𝒵}^{\left(t\right)}\right)\right\|}_{F}\leq M_{X}\cdot {\left\|{𝒵}^{\left(t+1\right)}-{𝒵}^{\left(t\right)}\right\|}_{F}.$$ (109)

Since it has been proven that $\left\{{𝒵}^{\left(t\right)}\right\}$ is a Cauchy sequence, the right-hand side is bounded.

On the basis of (109) and $\left\{{𝒴}^{\left(t\right)}\right\}$ is a bounded sequence, we have

$${\left\|{ℰ}^{\left(m\right)}-{ℰ}^{\left(n\right)}\right\|}_{F}\;\leq M_{X}\cdot {\left\|{𝒵}^{\left(t+1\right)}-{𝒵}^{\left(t\right)}\right\|}_{F}+\frac{M_{2}}{\mu_{2}^{\left(t\right)}}+\frac{1}{\mu_{2}^{\left(t\right)}}{\left\|{𝒴}^{\left(t\right)}\right\|}_{F}+\frac{1}{\mu_{2}^{\left(t-1\right)}}{\left\|{𝒴}^{\left(t-1\right)}-{𝒴}^{\left(t\right)}\right\|}_{F}\;\leq \sum_{t=n}^{m-1}\left(\frac{M_{X}\sqrt{N}\epsilon }{\rho^{t}\mu_{1}^{\left(0\right)}}+\frac{3M_{1}M_{X}}{\rho^{t}\mu_{1}^{\left(0\right)}}+\frac{4M_{1}M_{X}}{\rho^{t-1}\mu_{1}^{\left(0\right)}}+\frac{2M_{2}}{\rho^{t}\mu_{2}^{\left(0\right)}}+\frac{2M_{2}}{\rho^{t-1}\mu_{2}^{\left(0\right)}}\right).$$ (110)

Therefore, $\left\{{ℰ}^{\left(t\right)}\right\}$ is a Cauchy sequence.

Next, we prove that $\left\{{𝒬}^{\left(t\right)}\right\}$ is a Cauchy sequence. From the update ${𝒢}_{2}^{\left(t+1\right)}={𝒢}_{2}^{\left(t\right)}+\mu_{1}^{\left(t\right)}({𝒵}^{\left(t+1\right)}-{𝒬}^{\left(t+1\right)})$, this yields

$${\left\|{𝒬}^{\left(t+1\right)}-{𝒬}^{\left(t\right)}\right\|}_{F}={\left\|{𝒬}^{\left(t+1\right)}-\left({𝒵}^{\left(t\right)}+\frac{{𝒢}_{2}^{\left(t\right)}}{\mu_{1}^{\left(t\right)}}\right)+\frac{{𝒢}_{2}^{\left(t\right)}}{\mu_{1}^{\left(t\right)}}+\frac{{𝒢}_{2}^{\left(t\right)}-{𝒢}_{2}^{\left(t-1\right)}}{\mu_{1}^{\left(t-1\right)}}\right\|}_{F}\;\leq {\left\|{𝒬}^{\left(t+1\right)}-{𝒥}^{\left(t\right)}\right\|}_{F}+{\left\|\frac{{𝒢}_{2}^{\left(t\right)}}{\mu_{1}^{\left(t\right)}}+\frac{{𝒢}_{2}^{\left(t\right)}-{𝒢}_{2}^{\left(t-1\right)}}{\mu_{1}^{\left(t-1\right)}}\right\|}_{F},$$ (111)

where ${𝒥}^{\left(t\right)}={𝒵}^{\left(t\right)}+\frac{{𝒢}_{2}^{\left(t\right)}}{\mu_{1}^{\left(t\right)}}$.

The subproblem for 𝒬 is

$${𝒬}_{ijk}^{\left(t+1\right)}=\arg\min_{{𝒬}_{ijk}}\lambda_{1}{\left(M_{ijk}|{𝒬}_{ijk}|\right)}^{q}+\frac{\mu_{1}^{\left(t\right)}}{2}{\left({𝒥}_{ijk}^{\left(t\right)}-{𝒬}_{ijk}\right)}^{2}.$$ (112)

By Lemma 5, the optimality condition is

$$\lambda_{1}q{\left(M_{ijk}|{𝒬}_{ijk}^{\left(t+1\right)}|\right)}^{p}+\mu_{1}^{\left(t\right)}{𝒬}_{ijk}^{\left(t+1\right)}\left({𝒬}_{ijk}^{\left(t+1\right)}-{𝒥}_{ijk}^{\left(t\right)}\right)=0.$$ (113)

Note that ${𝒬}_{ijk}^{\left(t+1\right)}=0$ if and only if ${𝒥}_{ijk}^{\left(t\right)}=0$.

If ${𝒬}_{ijk}^{\left(t+1\right)}\neq 0$, then

$${\left({𝒬}_{ijk}^{\left(t+1\right)}-{𝒥}_{ijk}^{\left(t\right)}\right)}^{2}={\left(\frac{\lambda_{1}q{\left(M_{ijk}|{𝒬}_{ijk}^{\left(t+1\right)}|\right)}^{q}}{\mu_{1}^{\left(t\right)}{𝒬}_{ijk}^{\left(t+1\right)}}\right)}^{2}\leq {\left(\frac{\lambda_{1}M_{ijk}^{q}|{𝒬}_{ijk}^{\left(t+1\right)}{|}^{q-1}}{\mu_{1}^{\left(t\right)}}\right)}^{2}.$$ (114)

Since 0<q≤1 and $M_{ijk}\in [0,1]$, we have $M_{ijk}^{2q}\leq 1$. Moreover, by Lemma 5, there exists a positive lower bound δ>0 such that $|{𝒬}_{ijk}^{\left(t+1\right)}|\geq \delta$ when ${𝒬}_{ijk}^{\left(t+1\right)}\neq 0$, so

$$|{𝒬}_{ijk}^{\left(t+1\right)}{|}^{2q-2}\leq \delta^{2q-2}.$$ (115)

Let *M*_3_ be the upper bound in (115). Then for ${𝒬}_{ijk}^{\left(t+1\right)}\neq 0$,

$${\left({𝒬}_{ijk}^{\left(t+1\right)}-{𝒥}_{ijk}^{\left(t\right)}\right)}^{2}\leq \frac{\lambda_{1}^{2}M_{3}}{{\left(\mu_{1}^{\left(t\right)}\right)}^{2}}.$$ (116)

If ${𝒬}_{ijk}^{\left(t+1\right)}=0$, then ${𝒥}_{ijk}^{\left(t\right)}=0$, and the inequality holds trivially.

Therefore,

$${\left\|{𝒬}^{\left(t+1\right)}-{𝒬}^{\left(t\right)}\right\|}_{F}\leq \frac{\lambda_{1}\sqrt{n_{1}Nn_{3}M_{3}}}{\mu_{1}^{\left(t\right)}}+\frac{1}{\mu_{1}^{\left(t\right)}}{\left\|{𝒢}_{2}^{\left(t\right)}\right\|}_{F}\;+\frac{1}{\mu_{1}^{\left(t-1\right)}}{\left\|{𝒢}_{2}^{\left(t\right)}\right\|}_{F}+\frac{1}{\mu_{1}^{\left(t-1\right)}}{\left\|{𝒢}_{2}^{\left(t-1\right)}\right\|}_{F}.$$ (117)

Since $\left\{{𝒢}_{2}^{\left(t\right)}\right\}$ is bounded by *M*_4_ and $\mu_{1}^{\left(t\right)}$ grows geometrically, we have

$${\left\|{𝒬}^{\left(m\right)}-{𝒬}^{\left(n\right)}\right\|}_{F}\leq \sum_{t=n}^{m-1}{\left\|{𝒬}^{\left(t+1\right)}-{𝒬}^{\left(t\right)}\right\|}_{F}\;\leq \sum_{t=n}^{m-1}\left(\frac{\lambda_{1}\sqrt{n_{1}Nn_{3}M_{3}}}{\rho^{t}\mu_{1}^{\left(0\right)}}+\frac{2M_{4}}{\rho^{t}\mu_{1}^{\left(0\right)}}+\frac{M_{4}}{\rho^{t-1}\mu_{1}^{\left(0\right)}}\right).$$ (118)

Thus, $\left\{{𝒬}^{\left(t\right)}\right\}$ is a Cauchy sequence.

Finally, the proof that $\left\{{𝒯}^{\left(t\right)}\right\}$ is a Cauchy sequence follows similarly from the update ${𝒢}_{3}^{\left(t+1\right)}={𝒢}_{3}^{\left(t\right)}+\mu_{1}^{\left(t\right)}({𝒵}^{\left(t+1\right)}-{𝒯}^{\left(t+1\right)})$.

In conclusion, $\left\{{𝒞}^{\left(t\right)}\right\}$, $\left\{{𝒵}^{\left(t\right)}\right\}$, $\left\{{ℰ}^{\left(t\right)}\right\}$, $\left\{{𝒬}^{\left(t\right)}\right\}$, and $\left\{{𝒯}^{\left(t\right)}\right\}$ are all Cauchy sequences and hence converge to the critical points of the objective function ℒ(𝒵,𝒞,𝒬,𝒯,ℰ). ◻

Additionally, by running Algorithm 1 on the four datasets above, we practically validated the convergence of the proposed 2D-NLRSC. As shown in Fig 7, the error curves quickly stabilize after a few iterations on each dataset. These two aspects collectively demonstrate that the 2D-NLRSC model exhibits stability and rapidity.

### 6.1 Convergence rate analysis

We complement the convergence proof by quantifying the rates for both feasibility residuals and objective/value descent under standard assumptions used by augmented Lagrangian and splitting schemes.

**Notation 1.** Let the penalty parameters satisfy $\mu_{1}^{\left(t\right)}=\rho \;\mu_{1}^{\left(t-1\right)}$ and $\mu_{2}^{\left(t\right)}=\rho \;\mu_{2}^{\left(t-1\right)}$ with a fixed ρ>1. Define the primal feasibility residuals

$$r_{C}^{\left(t\right)}:=\|Z^{\left(t\right)}-C^{\left(t\right)}{\|}_{F},\;r_{Q}^{\left(t\right)}:=\|Z^{\left(t\right)}-Q^{\left(t\right)}{\|}_{F},\;r_{T}^{\left(t\right)}:=\|Z^{\left(t\right)}-T^{\left(t\right)}{\|}_{F},$$ (119)

and the data-consistency residual

$$r_{E}^{\left(t\right)}:=\|X-X*Z^{\left(t\right)}-E^{\left(t\right)}{\|}_{F}.$$ (120)

**Theorem 5** (Geometric decay of feasibility residuals). Suppose $\left\{G_{1}^{\left(t\right)}\right\}$, $\left\{G_{2}^{\left(t\right)}\right\}$, $\left\{G_{3}^{\left(t\right)}\right\}$ and $\left\{Y^{\left(t\right)}\right\}$ are bounded (as established by Lemmas 6–9). Then there exist finite constants $M_{1},M_{2},M_{3},M_{Y}>0$ such that, for all t≥0,

$$r_{C}^{\left(t+1\right)}\leq \frac{2M_{1}}{\mu_{1}^{\left(t\right)}},\;r_{Q}^{\left(t+1\right)}\leq \frac{2M_{2}}{\mu_{1}^{\left(t\right)}},\;r_{T}^{\left(t+1\right)}\leq \frac{2M_{3}}{\mu_{1}^{\left(t\right)}},\;r_{E}^{\left(t+1\right)}\leq \frac{2M_{Y}}{\mu_{2}^{\left(t\right)}}.$$ (121)

Consequently, since $\mu_{1}^{\left(t\right)}=\mu_{1}^{\left(0\right)}\rho^{t}$ and $\mu_{2}^{\left(t\right)}=\mu_{2}^{\left(0\right)}\rho^{t}$ with ρ>1, all residuals decay *R-linearly*

$$r_{•}^{\left(t\right)}=O(\rho^{-t})\;(•\in \{C,Q,T,E\}).$$ (122)

*Proof:* From the optimality of the C-subproblem and the update $G_{1}^{\left(t+1\right)}=G_{1}^{\left(t\right)}+\mu_{1}^{\left(t\right)}(Z^{\left(t+1\right)}-C^{\left(t+1\right)})$, we have

$$Z^{\left(t+1\right)}-C^{\left(t+1\right)}=\frac{G_{1}^{\left(t+1\right)}-G_{1}^{\left(t\right)}}{\mu_{1}^{\left(t\right)}}\;\Rightarrow \;r_{C}^{\left(t+1\right)}\leq \frac{\|G_{1}^{\left(t+1\right)}{\|}_{F}+\|G_{1}^{\left(t\right)}{\|}_{F}}{\mu_{1}^{\left(t\right)}}\leq \frac{2M_{1}}{\mu_{1}^{\left(t\right)}}.$$ (123)

The same argument using the Q and T updates yields the bounds for $r_{Q}^{\left(t+1\right)}$ and $r_{T}^{\left(t+1\right)}$. For $r_{E}^{\left(t+1\right)}$, the optimality of the E-subproblem together with $Y^{\left(t+1\right)}=Y^{\left(t\right)}+\mu_{2}^{\left(t\right)}(X-X*Z^{\left(t+1\right)}-E^{\left(t+1\right)})$ gives

$$r_{E}^{\left(t+1\right)}=\frac{\|Y^{\left(t+1\right)}-Y^{\left(t\right)}{\|}_{F}}{\mu_{2}^{\left(t\right)}}\leq \frac{2M_{Y}}{\mu_{2}^{\left(t\right)}}.$$ (124)

Geometric decay follows from $\mu_{•}^{\left(t\right)}=\mu_{•}^{\left(0\right)}\rho^{t}$. ◻

**Remark 1.** Theorem 5 implies that the KKT termination test in (92) is met after O(log(1/ε)) iterations, dominated by the largest of the four residuals.

## 7 Conclusion

This study proposes 2D-NLRSC, a submodule clustering framework using nonconvex low-rank tensor approximation. Unlike traditional vectorization approaches, it rotates 2D images into tensors and employs the *t*-product to derive self-representative tensors. The key innovations involve an $\ell_{r}$-induced tensor nuclear norm for optimal low-rank representation, along with a combined $\ell_{2,p}$-norm and $\ell_{q}$-norm regularization to enhance structure capture while effectively handling noise and redundancy. Theoretical convergence is demonstrated through the KKT conditions. Experiments on ORL, JAFFE, CMU-PIE, Yale, and MNIST show superior clustering accuracy and computational efficiency. Despite these advantages, this study still has certain limitations. The performance of 2D-NLRSC relies on the selection of several hyperparameters, which may require adjustment when applied to new datasets. Moreover, although relatively efficient, the computational complexity of its iterative optimization process remains relatively high for very large-scale image data. Future work will focus on automating hyperparameter selection and extending the framework to handle higher-order imaging data.
